# Changing the field: A Bourdieusian analysis of educational practices that support equitable outcomes among minoritized youth on two informal science learning programs

**DOI:** 10.1002/sce.21602

**Published:** 2020-10-28

**Authors:** Louise Archer, Spela Godec, Angela Calabrese Barton, Emily Dawson, Ada Mau, Uma Patel

**Affiliations:** ^1^ Institute of Education University College London London UK; ^2^ Educational Studies Department University of Michigan School of Education Ann Arbor Michigan USA; ^3^ University College London Science and Technology Studies London UK

**Keywords:** Bourdieu, equity/social justice, informal science learning (ISL)

## Abstract

Supporting more equitable participation in science, technology, engineering, and mathematics (STEM) remains a key, persistent educational challenge. This paper employs a sociological Bourdieusian lens to explore how equitable youth outcomes might be supported through informal science learning (ISL). Drawing on multimodal, ethnographic data from four case study youth aged 11–14 from two ISL programs, we identify four areas of practice that were enacted to a greater or lesser extent in the programs in support of equitable youth outcomes. We identify how the equitable potential of these practices was realized through a disruption of dominant power relations. It is argued that ISL should focus on changing the *field*, rather than young people. Affordances and limitations of the Bourdieusian lens are discussed.

## 1 EQUITY AND INFORMAL SCIENCE LEARNING (ISL)

The ISL sector comprises a plethora of organizations, settings, and initiatives, operating across a wide range of scales and budgets. It includes designed spaces (e.g., science centers, museums), community spaces (e.g., clubs, local organizations), events (e.g., festivals, science cafes, citizen science), award schemes (e.g., competitions), and everyday forms of engagement (e.g., science‐related TV, internet, and media). It is funded and delivered by a diverse range of organizations, ranging from the national government, to professional societies, educational institutions, community groups, campaign groups, industry, and the private sector. In short, ISL constitutes a significant area of educational activity and—due to prevalent national economic imperatives to address the skills gap in STEM (science, technology, engineering, and mathematics)—occupies a position of strategic policy importance (e.g., Nath & Collins, 2011).

It has been argued that ISL settings can provide a way in, or “on‐ramp,” to STEM for young people, especially those who have had negative or unengaging experiences of school science (e.g., Falk & Dierking, 2010; McCreedy & Dierking, 2013; Calabrese Barton and Tan, 2010). Proponents suggest that ISL offers an alternative space for science learning, free from the perils of performativity and accountability that characterize formal education (Stocklmayer et al., 2010). Accordingly, many ISL spaces are characterized by a mission to inspire and inform young people about science. Yet, in general the potential for ISL to engage diverse young people with science remains unfulfilled, as evidence shows that most ISL participants tend to come from socially privileged, white, affluent backgrounds (Dawson, 2019; Feinstein & Meshoulam, 2014), reflecting the characteristics of those who tend to pursue post‐compulsory science within the formal education sector (e.g., Smith, 2011). Moreover, attention has been drawn to the ways in which access to ISL is structured by social inequalities and how the culture of ISL spaces tends to represent, value, and reproduce dominant, white, male, middle‐class values, histories, and identities (e.g., Archer et al., 2016; Borun, 1999; Crowley, 1999; Crowley et al., 2001; Dancu, 2010; Ramey‐Gassert, 1996).

Equity can, and has been, conceptualized in a range of different ways in relation to education and ISL. In this paper, we take the position that ISL participation is valued as a way to position youth and communities with agency and authority—not just to serve the science pipeline. Equity work thus requires challenging the power structures which create inequalities in, and inhibit, participation.

### 1.1 Equity‐focused research in ISL

There is an important body of innovative, equity‐focused research and development work conducted in ISL contexts—but particularly in longer‐term afterschool club programs—that suggests that some ISL spaces can and do successfully engage and support young people from traditionally underrepresented communities to develop an identification with science and achieve expansive outcomes. Examples include, for instance, Calabrese Barton and colleagues' GET City initiative (e.g., Calabrese Barton & Tan, 2010), the Fifth Dimension program (e.g., Cole & The Distributed Literacy Consortium, 2006), and the Computer Clubhouse (e.g., Resnick & Rusk, 1996), to name but a few. Reviewing the critical literature on equitable youth participation in ISL, we identified four key common principles and approaches to practice in support of equitable youth outcomes: (i) centering youth—framing ISL through the identities, values, and experiences of youth; (ii) challenging elite STEM practices, epistemologies, and representations; (iii) supporting young people's critical STEM agency; and (iv) respecting and valuing young people's identities in STEM. These are now discussed briefly in turn.

#### 1.1.1 Centering youth: Framing ISL through the identities, values, and experiences of youth

There is an established critical tradition of grounding ISL in the identities, experiences, values, and what matters to young people. This practice seeks to recognize and value youth as not just consumers but also producers of STEM. It also seeks to reframe STEM in more inclusive ways, connecting with and through young people's lives to enable consequential STEM learning that matters (e.g., Adams & Gupta, 2013; Birmingham and Calabrese Barton, 2014; Birmingham et al., 2017; Calabrese Barton & Tan, 2018; Habig et al., 2020; Tan & Calabrese Barton, 2020).

#### 1.1.2 Challenging elite STEM practices, epistemologies, and representations

The critical literature argues that to support equity, ISL experiences need to engage with and reflect on *whose* and *which* (STEM) knowledge and practices are valued and represented (e.g., Medin & Bang, 2014), disrupting dominant versions and prioritizing more equitable and inclusive epistemological approaches and stances (e.g., Bang & Vossoughi, 2016). This practice involves ISL spaces and programs explicitly challenging and reworking elite cultural discourses and practices in/of STEM (e.g., Chaffee & Gupta, 2018) and supporting youth to challenge and expand ways of being in ISL spaces (e.g., Calabrese Barton & Tan, 2010; Calabrese Barton et al., 2008; Medin & Bang, 2014; Rahm, 2010; Thompson, 2014).

#### 1.1.3 Supporting young people's critical STEM agency

The critical ISL literature foregrounds the value and importance of not just supporting science‐orientated goals—such as young people's science learning and/or science‐related pathways—but supporting young people's critical STEM agency. Critical STEM agency refers to young people using STEM practices and knowledge to take action on issues that they personally care about (e.g., Adams & Gupta, 2013; Birmingham et al., 2017; Calabrese Barton & Tan, 2010; Calabrese Barton et al., 2008; Medin & Bang, 2014; Rahm, 2010; Restrepo Nazar et al., 2019; Thompson, 2014; Tan & Calabrese Barton, 2020). This can involve supporting youth to challenge dominant master narratives in STEM and break down hierarchical binaries of what knowledge, experiences, and voices matter in STEM (Birmingham et al., 2017).

#### 1.1.4 Respecting and valuing young people's identities in STEM

Research shows how young people can be supported to develop science identities when they have opportunities to leverage their lived experiences and community wisdom as an integral part of doing science and engineering—and when they receive recognition for this (Calabrese Barton & Tan, 2018). Emphasis is placed on the value of assets‐based and participatory approaches in ISL, which seek to respect and value youth and community knowledge and resources. Such approaches stand in opposition to damage‐centered research (Tuck, 2009), that is, research which only focuses on the damages and injustices done to and experienced by minoritized youth.

The critical literature thus points to four key areas of equitable practice for ISL settings which, as discussed further below, we bring into dialogue with a Bourdieusian lens. We do this to learn more about how and why particular ISL practices and enactments of these principles and practices might support particular equitable youth outcomes in different programs and the extent to which such practices might (or might not) be transferable and travel over time and space within and between settings. This endeavor is especially urgent in the context of designed settings, not least because in these spaces “the things that people *do* to give meaning to the concept of inclusion (Florian, 2009) is not well articulated” (Florian & Black‐Hawkins, 2011, p. 814).

We seek to add to understandings of relationships between ISL practices and the achievement of equitable youth outcomes, exploring the extent to which specific enactments of practice (aligned with the four practices discussed above) were evident, or not, within two reasonably typical ISL settings—a community space and a designed ISL setting—through the lens of four focal youth. By equitable youth outcomes we mean those that move toward equitable and transformative outcomes for young people, disrupting the social reproduction of inequalities in some way, either as part of a young person's experience on a program, or in some more sustained or general way that endures beyond the program experience. To explore these ideas, we apply a Bourdieusian lens to empirical data collected with youth aged 11–14 who participated in two ISL programs in London, UK, one in the designed space of a zoo and the other in an afterschool STEM club.

### 1.2 A Bourdieusian conceptual lens

A Bourdieusian analytic lens can be useful for understanding and identifying ISL practices that support equitable youth outcomes in the programs. We use a Bourdieusian approach because it is centrally concerned with the production and nature of practice and foregrounds structural inequalities and relations of power, making it apt for engaging with issues of injustice. In addition, Bourdieusian theory has not yet been extensively applied to the topic of ISL participation, although our own previous work has suggested it may be able to offer new insights (cf. Archer et al., 2016; Dawson, 2019).

Bourdieu's extensive body of work has been applied predominantly within the field of formal education, notably compulsory and higher education. This study underlines the key role that the education system plays in the social reproduction of relations of privilege and domination, as most notably explicated in *Reproduction in Education, Society and Culture* (Bourdieu & Passeron, 1990). At its heart, Bourdieu (e.g., 1977) proposes that social reproduction is produced through the interaction of *habitus* (embodied, socialized dispositions which are both structuring and structured) and *capital* (economic, social, and cultural resources) within *field* (socio‐historical spaces of positions and position‐taking, each with a logic of practice, that sets the “rules of the game” and determines who/what is valued within that setting).

Habitus refers to the embodied, socialized framework of dispositions, which provides our practical feel for the world. Cultivated particularly through experiences at home and school, habitus is structured by the social world and our experiences in it and, in turn, structures our perceptions and interactions with the world. Habitus is not just in the mind but is also embodied through bodily gestures, accent, and ways of being, as encapsulated by the notion of taste (Bourdieu, 1986, p. 6). Habitus operates in conjunction with capital—which refers to the economic, cultural, and social resources that a person possesses. The nature, volume, and types of capital that someone has will be shaped by the field, which determines the value of capital and the potential for its accrual (Bourdieu, 1986, p. 242).

Field refers to the socio‐spatial configurations of power relations which set the rules of the game within a given arena. Field is more than just a context—it can be understood as an arena of power relations and positioning (Bourdieu, 1990a). While fields are bounded to an extent, they also overlap and work across different scales and levels. For instance, we might consider the field of ISL to comprise many overlapping subfields—including designed, community, and everyday ISL fields, the field/s of science and STEM, the field of a specific institution, program, and so on. Hence each ISL subfield will have its own particular logic of practice, rules of the game and configurations of social actors who are located in symbolic space through differential power relations. While field is a necessarily elastic concept, it is also useful for our purposes as it foregrounds power relations and understands young people's ISL experiences as generated through the interaction of what young people bring with them (habitus and capital) with the multiply configured power relations that structure any given setting (field). That is, it facilitates an analysis of why young people may have differential experiences and outcomes from participation in the same ISL program/setting.

Bourdieu did not apply his theory to the context of ISL per se, although he did address inequalities in museum participation. Writing in the 1960s in the context of European art museums, Bourdieu and Darbel (1997) explained how societal inequalities mean that nondominant communities may not acquire a taste for museums. Moreover, when these communities do visit, they are often Othered and disadvantaged in these spaces because they do not possess the “right” (i.e., dominant) cultural ciphers to unlock the meaning of particular museum artefacts. This study has been influential within museum studies as it has been argued that Bourdieusian theory “offers a powerful way of theorizing the museum–visitor relationship” (Dicks, 2016, p. 52). While Bourdieu wrote predominantly from the perspective of social class, more recent research has drawn attention to how designed ISL spaces, like science museums, tend to privilege dominant, western, and male forms of doing and knowing and fail to serve and support people from minority ethnic and working class groups, who are excluded and marginalized by the practices, norms, and expectations within these spaces (e.g., Archer et al., 2016; Dawson, 2014).

Our Bourdieusian lens thus explains how youth are Othered and made to feel out of place in many ISL settings due to a disjuncture between habitus and field and because the field is set up to further the interests of the dominant. It also draws attention to how Other young people are disadvantaged because their habitus and capital are negated and/or positioned in deficit ways in the ISL field. Hence, while ISL settings may purport to welcome diverse youth, the dominant norms and power relations in these fields mean that participation is contingent on youth acquiring the “right”—that is, dominant—forms of capital that can be valued and leveraged in support of participation. These practices marginalize youth and also hide the inequitable power dynamics that perpetuate systemic injustices, which are rendered invisible because they are normalized through the everyday workings of ISL settings as just the way things are—a state that Bourdieu terms *doxa*. As discussed above, it has been argued that to date, dominant approaches to widening participation in ISL have only minimally disrupted the racial, gendered, classed, and linguistic hierarchies in informal science education, while mostly maintaining these oppressive power dynamics. Applying our Bourdieusian lens to ISL helps us to understand how and why these relations persist and are continually re/produced.

#### 1.2.1 Bringing the Bourdieusian lens into dialogue with the critical ISL literature

There are three key ways that the critical ISL literature informs and extends the Bourdieusian lens that is adopted in this paper. First, the critical literature brings a precision and focus on the ISL context, along with explication of the sorts of practices that support equity and social justice for youth in these settings—aspects that do not exist in Bourdieu's original work. Notably, the critical literature showcases some “ideal type” ISL programs (such as GET City) that are not typical of the wider ISL field and which provide spaces of inspiration and possibility for the sector. Second, the critical ISL literature foregrounds agency, which although present in Bourdieu's work, is not articulated as strongly as his focus on structure. Third, the critical work engages directly and productively with dimensions of race and gender, directly addressing well‐known areas of weakness in Bourdieu's work.

We bring these contributions from the critical literature into dialogue with the Bourdieusian lens to enhance the application of the lens to the ISL context and professional practice, and to extend the equitable potential of the lens. We use the lens to explore and understand the extent to which the practices identified in the critical literature were present (or not) in the programs that we researched, paying particular attention to the ways in which youth agency was supported, or not. Combining the lens with the ISL literature also enables us to identify and understand not only the classed dimensions of the young people's experiences and outcomes, but also the gendered and racialized aspects.

Just as the critical literature augments and extends the Bourdiesian lens, the sociological framework also offers three main contributions to existing work on equity in ISL. First, Bourdieu offers a compelling account of how and why it is no accident that minoritized youth are—and continue to be—marginalized, excluded, and oppressed in education, STEM, and ISL fields. The Bourdieusian lens also explains why attempts at innovation and reform of mainstream ISL practice may be difficult to enact and resistant to change. Second, Bourdieu's work offers a “grounding” of agency (Bourdieu & Wacquant, 1992), explaining how agency is not some ephemeral or amorphous quality but involves multiple forms of capital and power (Bourdieu, 1986) and identifies the locus for change as lying in the interaction between field and habitus (Lawler, 2011). Thus, using the Bourdieusian lens, we understand that enacting equitable practice will depend heavily upon the interaction of habitus, capital, and field (the organization, power relations, positionality, values, structure, culture, and actors within a given ISL setting or program). These interactions will shape how practices are interpreted and enacted and the outcomes for youth. A Bourdieusian lens foregrounds how practices are not abstract, universal, or discrete phenonomena but are highly localized and specific products of temporal and socio‐spatial relations, namely configurations and interactions between field(s) and the habitus and capital of the social actors in question.

Third, the Bourdieusian lens emphasizes the importance of processes of *conversion* within the four approaches identified in the critical ISL literature. It helps explain how and why it is important to value youth identities, assets, experiences, and ways of knowing/being, showing that while such practices may be enacted at a personal, individual level, these practices are simultaneously orientated toward social transformation, seeking to fundamentally disrupt the hidden practices and structures through which dominant, oppressive social relations are re/produced and maintained. From this perspective, we interpret the four practices outlined in the critical literature as “undoing” what Bourdieu terms *social magic* (Bourdieu, 1991), transforming the production of *symbolic capital*.

Social magic is “the means of obscuring the conditions in which value is constructed so that it comes to be seen as ‘natural’ and the cultural arbitrary is denied” (Ingram & Allen, 2019). Bourdieu uses the term to refer to the way in which arbitrary forms of embodied capital are converted into socially valued forms so that the context of their original production is hidden and they appear as “natural” traits, talents, or attributes of the (privileged) person in question. Through this process, particular attributes come to symbolize particular qualities (such as certain masculinized, middle‐class behaviors coming to be recognized as signifying an “authentic science student,” e.g., Archer et al., 2018). Through social magic, “symbolic capital disguises its own status as a social product” (Lawler, 2011). As Steph Lawler (2011) explains, “symbolic capital is not a different form of capital, but rather should be seen as the legitimated, recognized form of the other capitals,” namely economic, cultural, or social capital. The process of legitimation is important because not all forms of capital have equal value and weight. Hence “only some capitals 'count' […] and this counting is an outcome of symbolic struggles in which some groups have acquired the power to name specific types of the capitals as legitimate” (Lawler, 2011).

In this way, we interpret the approaches identified by the critical literature (centering youth, challenging elite STEM representations, supporting young people's critical STEM agency and respecting and valuing young people's identities in STEM) as seeking to disrupt dominant processes of the legitimization of capital, enabling marginalized and oppressed forms of habitus and capital to be converted into symbolic capital—although this recognition is often largely restricted to the ISL field in question and the academic context. By troubling the dominant ways in which symbolic value is accorded within ISL, critical approaches help to pull back the curtain to reveal the social magic at work that naturalized the experiences and attributes of privileged young people that results in most ISL being the preserve of the white, middle‐classes.

Bourdieu focuses on identifying how social magic and symbolic capital play a key role in social reproduction. However in this paper, we explore how re‐working these processes—that is, changing the field to shift what/who gets valued in ISL—might provide a potential means for social transformation. While Bourdieu did not explore this potential, we suggest that his ideas can be extended to show that the liberatory potential of the four approaches discussed in the critical ISL literature derives from their reworking of the processes and relations through which social inequity is re/produced, namely undoing social magic and transforming what and how capital is legitimated.

#### 1.2.2 Theorizing practice, outcomes, and the practice/outcomes relationship from a Bourdieusian perspective

In this paper, we explore enactments of ISL practices through the lens of four focal youth, tracing the extent to which these enactments might support equitable youth outcomes. Accordingly, we now set out how, through our theoretical framework, we conceptualize practice, equitable youth outcomes and the relationship between the two. We understand practice as “a temporally and spatially dispersed nexus of doings and sayings” (Schatzki, 1996, p. 89), which comprises material, social, symbolic and discursive elements, competencies, and performances (Shove et al., 2012) by a range of actors, resources, and institutions within and across different fields. Or as Wetherell (2012) puts it, “practice is both a noun and a verb” (p.23)—comprising both specific elements and “stuff” (e.g., material, social/cultural and symbolic actions, and resources/capital) and performance. Moreover, practice entails both improvisation and training, the latter of which we understand as a form of Bourdieusian socialization that can be achieved by a range of formal or informal institutions.

We understand *outcomes* as complex, multiple, contradictory, cross‐cutting, provisional phenomena that are always in process and are challenging to measure. That is, outcomes are not fixed, discrete, or neatly achieved phenomena. Our understanding of what constitutes an *equitable outcome* for a young person draws on wider conceptual work that we conducted in the project over a 3‐year period, which led to the production of a model that synthesizes across “traditional” (mainstream), critical and sociological approaches to ISL research and evaluation, which produced a model of equitable youth outcomes that include four key areas: STEM capital, STEM identity, STEM trajectories, and (STEM) Agency (see [anonymised reference] for full details). In this paper we interpret these four outcome areas through a Bourdieusian lens, in which an equitable outcome from ISL is one that results in:

- the disruption and restructuring of inequalities in STEM capital, representation, and trajectories for a young person and/or their community;
- support, enhancement, and meaningful benefit to the (STEM) habitus (identity), capital and agency of young people from under‐served communities.

While we recognize that there may be a wide range of potential outcomes from ISL participation, these may not necessarily be equitable and, following Bourdieu, many are likely to be in the service of the social reproduction of inequalities. Indeed, from this perspective, we would argue that outcomes are either equitable or inequitable—but they are not neutral, because the status quo, or default, is the reproduction of inequalities. Hence, equitable outcomes must necessarily entail a challenging of the status quo.

We understand that equitable outcomes can occur across a considerable range of scales: from shifts in representation, capital and habitus in the moment of a particular ISL program through to more sustained or generalizable changes that might alter, for instance, how a young person feels about themselves or is recognized by others in other settings, such as school science. The most substantial equitable outcomes would relate to significant transformations in young people's social and economic well‐being and changes to the wider fields of STEM and ISL. The majority of outcomes that we discuss in this paper were modest and restricted to the field of young people's participation in a particular ISL program. We did not identify any examples of these outcomes extending to wider or longer‐term changes, such as the impact on young people's experiences of school science and/or their later lives. Indeed, our field work only extended to following young people up 5–6 months after the end of the programs. We thus focus predominantly on outcomes that related to young people's experiences within the two ISL programs and to a lesser extent some wider outcomes, largely relating to changes in habitus, personal confidence, and agency, that some young people identified during follow‐up interviews.

Our understanding of the *relationship between practice and equitable youth outcomes* is informed by Bourdieu's proposal that habitus and practice exist in recursive relation, such that habitus is “constituted in practice and is always orientated towards practical functions” (1990b: 52). However, we do not assume that the relationship between practice and outcome is straightforward, linear, or causal. In other words, we do not think that performance of a particular practice will necessarily result in or produce a particular outcome. Rather, we understand practice as being constructed through enactments that are performed across time and space and which are mediated by fields and by interaction with youth habitus and capital. In other words, the potential impact of an ISL practice on youth outcomes will be mediated by other factors and interrelationships (e.g., Shove et al., 2012). In particular, using a Bourdieusian lens, we understand that relations of power in the field will be particularly important in shaping what (ISL) practices are possible, how they are enacted and the ways in which interactions of habitus, capital, and field will mediate the outcomes experienced by young people who encounter and are subject to these practices.

We propose that the achievement of equitable youth outcomes from ISL can thus be understood as produced by a dialectical process, generated through interactions between what young people bring with them (habitus, capital) and the ISL field. Hence, there is no simple, linear process whereby a particular ISL practice will produce the same outcome in all participating youth, as this will be mediated by interactions between each unique habitus, capital, and field in a particular time and space (such as from their previous experiences). However, there may be broad, discernible patterns in terms of shared, collective aspects of habitus and capital in relation to institutionalized features of a field. For instance, where a field values and recognizes nondominant forms of capital, we might reasonably expect to see improved outcomes for minoritized youth whose capital and habitus are, respectively, leveraged and recognized.

#### 1.2.3 Issues and limitations of using a Bourdieusian lens to investigate equitable youth outcomes from ISL

We seek to identify and understand what configurations and practices within an ISL field might support young people to achieve equitable outcomes and have set out reasons, above, as to why a Bourdieusian lens might be appropriate for this task. However, while Bourdieu's work has been used extensively to understand the reproduction of dominant relations of inequality through education—how practices “congeal and constrain, producing difficult to shift social formations, hierarchies, epistemic regimes and patterns of distinction” (Wetherell, 2012, p. 23)—it has been used far less as a tool for examining how injustices might be challenged and transformed. This may not be surprising given that while it offers a compelling account of social reproduction, Bourdieu's theory has been critiqued for its pessimism and determinism, with the argument being made that it tells us relatively little about how and why change and transformation might be possible (e.g., Gunter & Willmott, 2002). In particular, it has been argued that Bourdieu's theory fails to explain instances when the dispossessed succeed against the odds, such as how minoritized youth might derive consequential outcomes from participation in ISL. The irony of this silence is underlined by Bourdieu's own biography and personal trajectory of social mobility, coming from a working‐class family and rising to the heights of academia, apparently thanks to his educational experiences and attainment. That is, curiously, Bourdieu's own biography was “at odds with his theory” (Burawoy & Van Holdt, 2012, p. 11).

At one point in his writings, Bourdieu entertained the potential for more transformative professional pedagogy, encapsulated in the notion of “rational pedagogy” as an imagined practice (“still to be invented”) for achieving equitable social change (Bourdieu & Passeron, 1979), in which education is used to systematically redistribute symbolic capital, such that initial inequities in the distribution of capital across social classes, are redressed. However, he subsequently came to regard this as an impossibly utopian ideal, arguing that as long as the class structure remains, it will pervert and constrain all attempts at redistribution. In this respect, Bourdieu's theory appears, at face value, to offer little to the critical researcher or pedagogue who wishes to challenge inequalities in and through their practice.

Some sociologists of education have argued that the accusation of determinism is overplayed and that Bourdieu's work can be extended to account for social change (e.g., Mills, 2008). For instance, Reay et al. (2009) study of educationally successful working‐class students studying at elite British universities highlighted how the students' encounters with the new, unfamiliar field of elite HE led to aspects of the habitus being restructured and transformed. Similarly, Yang (2014) sets out how a closer and more innovative reading of Bourdieu reveals several conditions for habitus change. In line with Bourdieu's original formulation, however, this study tends to foreground transformation at the level of the individual reflexive habitus, such that change within the field is understood as produced (only) through the actions of individuals, rather than educational settings or practitioners. In contrast, this paper seeks to contribute to the advancement of thinking with regard to this gap, applying a Bourdieusian lens to our empirical data, to identify what pedagogical practices and sets of relations within these settings might enable/support and promote the achievement of equitable outcomes (and transformed habitus and capital) for minoritized youth. Moreover, our interpretation of Bourdieu reminds us that capital in and of itself does not have an inherent value, rather the value is determined by the field and that *hysteresis* (the disjuncture between habitus and field) is not a permanent state but is contingent upon the field in question. Hence, we note that an educational field, as a space of forces, will also contain the potential for change in that it represents a space of possibility for different power relations, rules, habitus, and capital to “count.” In this respect, we propose that the lens is appropriate and that this relatively novel application—investigating the potential for ISL practice to support equitable youth outcomes—may offer potential new insights for the fields of both ISL and sociology of education.

To summarize, through application of a Bourdieusian lens to empirical data from four focal youth, this paper asks:

- What were the young people's experiences and outcomes from participation in and during the focal ISL program?
- In what ways did particular enactments of ISL practice support equitable youth outcomes within the programs? And in what ways could the programs have been more effective in supporting equitable youth outcomes?

## 2 METHODS

The data presented in this paper come from the Youth Equity and STEM (YESTEM) project, a 4‐year collaboration (funded by the NSF, Wellcome Trust, and Economic and Social Research Council) seeking to understand, develop, and support equitable practice within ISL programs. This paper draws only on data from the UK side of the project that was collected during the first year of the collaboration. During this time, the UK research team observed four programs run by ISL organizations, two of which are presented here (both based in London, UK). A summary of the focal programs, participants, time‐scale, and indicative content is provided below in Table 1, with a fuller description and detail in the Appendix.

**Table 1:** Details of the settings, programs, and young people

Setting	Program	Young people's self‐identifications	Summary of program
Community Zoo	One‐week holiday program	4 boys, 5 girls	One week, all day holiday program. Participants learned about habitats and ecology. They created portfolios, made artefacts for the zoo (e.g., bird boxes, signage, enrichment objects for enclosures) and participated in feeding animals and observing/recording and managing animal data.
		Black Caribbean (1), Middle Eastern (1), Mixed South Asian/White European (1), Mixed White European/North African (1), White British (5)	
		Working‐class (4), Middle class (5)	
Girls STEM Club	Weekly school‐based STEM club with industry visits (September–January)	10 girls	Weekly, 2‐h after‐school club with weekly themed sessions that showcase women in STEM (especially BAME). Participants choose from a carousel of STEM games and activities (e.g., making and flying paper airplanes, modeling the solar system). Also includes a school visit industry day (held at a City tech consultancy firm, where girls learn to code and create their own festival app) and a hackathon weekend (learning to program in Scratch and using Python to create a chatbot)
		Black British (5), Southeast Asian (1), White British/Irish (3), Mixed Black Caribbean/White British (1)	
		Working‐class (10)	

Abbreviations: BAME, Black, Asian and minority ethnic; STEM, science, technology, engineering and mathematics.

The first program was a week‐long nature‐focused science club held during the school holidays at a local community zoo. The program used a mixture of approaches and activities, typically involving a short introduction from Cole, the lead practitioner, followed by group discussion and a practical activity. Each day was split into three or four main sessions (morning and afternoon) that usually involved hands‐on, creative tasks relating to the natural world, habitats, and ecology. For instance, youth made enrichment objects for the animal enclosures, observed animal behaviors in the zoo, made personalized bug hotels for the zoo grounds, and created signage for visitors to explain the animals, their behaviors, and habitats. The theme of environmental sustainability, environmental threats, and care for the natural world was threaded throughout the program.

A more detailed plan is provided in the Appendix, but the following abbreviated field notes from the morning session from Day 3 gives a flavor of the program and approach.

The young people arrive and begin finishing off some self‐portraits that they had begun the day before (representing who they are and things that they feel are important about their identities and interests). Cole, the lead practitioner, asks everyone what are their favourite animals? The young people enthusiastically share and compare their favourites as they finish the portraits. The next session, an introduction to reptiles and parasites, begins with Cole asking the young people what reptiles they know. He congratulates each response and writes the contributions on the whiteboard. He explains which of these are in the zoo. He asks the youth for their ideas on how the zoo can keep the animals healthy and happy? The youth offer lots of suggestions that Cole values and scaffolds to help them think about how these relate to their own ways of keeping healthy. Cole asks what foods they like to eat and during the discussion shares that he is a vegetarian. The group talk about how humans are omnivores and identify animals that eat other animals. Cole asks what parasites they can name—the young people share their knowledge and experiences (e.g., Lulabelle says she has seen a TV programme about “a fish that cleans sharks teeth”). Cole hands out photos of different hosts and parasites for the group to match up. Lulabelle calls out that people are parasites which Cole uses as part of a discussion about parasites and parasitic behaviour. Charlie suggests that a cuckoo might be a parasite because she knows that “they lay their eggs in other birds' nests.” Lulabelle tells everyone that her road is named after one of the parasites that they are discussing. The group are very animated and spend some time discussing and sharing their ideas about hosts and parasites. After a short break, the next session focuses on animal diets and nutrition. Cole introduces the session by asking the young people about their own diets and why a balance (e.g., of carbohydrate, sugars, protein, etc.) is important. Evie and Lulabelle volunteer their own knowledge of the importance of balancing sugar intake for family members with diabetes, which Cole values as important expertise. Their next task is to feed the animals—they go out in small groups to the zoo kitchen to cut up vegetables and collect tubs of worms. They have to calculate how much what weights of carrots and worms will be needed for the pigs' lunch. Cole explains that this is a task that zoo staff have to do every day. He explains what factors they will need to work into their equations. The youth work as a group and struggle a bit to formulate their equations to work out the food weights. After a bit, Star strides up to the front and begins writing out the calculations for the group on the whiteboard. They work out the feed weights, then go to the kitchen to weigh the food before going out with Cole to feed the pigs.

Young people recorded their learning over the course of the week in individual portfolio scrapbooks, which they also took home to add to and work on further as “homework.” Each day was structured to include some classroom work, some creative activities, and some activities out in the zoo.

The program was attended by nine young people, seven of whom were recruited via a local school, which was asked to put forward a group with at least half of the students being eligible for free school lunches and from minority ethnic communities. This was to include young people who might not typically take part in such programs, which tend to be dominated by white, middle‐class young people, as also confirmed by the zoo practitioners. The sessions were led by Cole, who described himself as a mixed‐race, British, gay man from a working‐class background.

The second program was a weekly afterschool STEM club run by a social enterprise working with young women. In addition to weekly club sessions, the girls took part in two off‐site whole day sessions—a coding development day trip hosted at a global consultancy firm and a Saturday coding day held at a global bank. The club sessions followed a common format each time, beginning with a focus on a female STEM professional, such as celebrated mathematicians, computer scientists, and engineers. This was followed by a choice of two activities—either a making (creative) activity or an exploring activity, in which the young women would research a topic further on the internet and then present their findings back to the group (typically using PowerPoint slides). At the end of the session, members were asked to fill in a short evaluation survey. The following annotated field notes, from session four, provide a flavor of the club sessions:

The girls come in and help themselves to the selection of snacks and drinks. Maddison welcomes everyone, gives out the handouts for today's session and plays a short YouTube video about Nike Folyan, a Black British female chartered engineer, who talks about her career, experiences and reasons why she became (and enjoys being) an engineer. Maddison then explains the ‘make’ and ‘create’ activities for today. Most of the girls choose the ‘make’ activity, designing and flying paper airplanes. Maddison and Bobbi encourage the girls to think about refining their designs between flying rounds to improve the aerodynamics—although many girls just make a similar plane each time. They laugh and ask each other to film their flights and compete to fly theirs the furthest. Innocent and Dani choose to do internet research to make an infographic about Nike Folyan, and chat animatedly with one another about goings on in their friendship groups while they make two short powerpoint slides. Pop music plays in the background and the girls continue to chat and help themselves to snacks as they work. The session overruns a bit and there is no time for Innocent and Dani to share their research with the group. Innocent announces that she is leaving. Bobbi notices that Innocent seemed a bit bored or disengaged with the set task and goes over to talk to her. Bobbi asks Innocent if she would like to lead the session next week? Innocent appears pleased and Bobbi says, “OK so we have a deal?”

The 10 girls in the club were from the same central London school; seven were from minority ethnic communities and most were working‐class, as suggested by their parents' occupations. The clubs were predominantly run by Madison, who self‐identified as a White, lower middle‐class, young British woman who had recently graduated from university. One session was jointly run by Bobbi, the organization's director, who self‐identified as a Black British African, working‐class woman. All young people who took part in the ISL program agreed to participate in the study.

The research team collected data through observations, repeated semi‐ and unstructured interviews with youth and practitioners during and after the program, multimodal portfolios (e.g., selected photos and reflections on the program) and follow‐up discussion groups with young people using outcomes cards classification exercise. Details are provided on each form of data collection in Table 2. The multimodal data collection generated rich accounts, which were further complemented with researchers' reflective notes guided by the study's aims. All names mentioned in this paper are pseudonyms, which young people had chosen themselves.

**Table 2:** Details on data collection

Data collected	Participants	Description
Observations	Young people practitioners	Detailed field notes (taken by hand or using laptops/iPads) of what happens during each program session. Each session is recorded by two or more researchers (each of whom focus in depth on specific participants) who then combine and compare field notes to produce one agreed set for each field work session.
Interviews and discussion groups	Young people practitioners	2 × semi‐structured interviews with each young person (one during the program, one several months after the program)
		Discussion groups with 3–5 young people per group, conducted a couple of months after the end of the program to reflect on program experiences and outcomes
		Unstructured more informal chats with participants while they take part in the programs (e.g., during lunch breaks, while making objects during activities)
		Semi‐structured interviews with each practitioner during and after each program
Portfolios	Young people	Multimodal portfolios created by the young people, organized under common section headings, containing written text, drawings, photographs, videos, collages detailing their relationship with STEM, their interests, identities, and aspirations and their experiences of the program (what they liked, did not like, etc).
Outcomes card sort task	Young people	Conducted during the post‐program discussion group. Young people are given a set of cards with potential outcomes written on (relating to, e.g., how much they enjoyed the program; gained new knowledge and skills; changed their views of STEM, felt empowered by the program; did new things or made changes in their daily lives as a result of the program; felt that the program had broadened ideas of who does science), which were used as prompts for group discussion, exploring areas and reasons for agreement/disagreement.

Abbreviation: STEM, science, technology, engineering and mathematics.

We began data analysis by mapping young people's reported and observed *experiences* of the programs (see row 3 of Table 3). From this mapping, we identified two focal youth per setting, who recorded the most positive experiences in each setting (Lulabelle in the zoo and Crystal in the STEM club) and the most mixed experiences (Star in the zoo and Innocent in the STEM club). We next undertook a mapping of *youth outcomes*, which involved working across the different multimodal data for each youth, identifying outcomes in relation to four key areas: STEM‐related capital (STEM‐related knowledge, competencies, skills, dispositions, behaviors, and social contacts), STEM identity work, STEM trajectories (including aspirations to continue with STEM) with the last capturing more expansive outcomes relating to individual and collective agency. These four outcome areas had been previously identified as important dimensions of equitable youth outcomes, based on a mapping and synthesis of three literatures (traditional, critical, and sociological research on ISL), carried out within the wider project ([anonymised reference]). This analytic process resulted in a set of mapped outcomes for each young person—the key equitable outcomes for each youth are detailed rows 4.1–4.4 of Table 3.

**Table 3:** Summary of youth, outcomes, and practices

	Lulabelle	Star	Crystal	Innocent
Program	Zoo	Zoo	STEM club	STEM club
1. Self‐identification	White British working‐class girl	North African/European working‐class boy	White British/Irish, working‐class girl	Black African British, lower middle‐class girl
2. Pre‐program STEM attitudes, identity, and aspirations	Ambivalent STEM identity, no science aspirations	Strong STEM identity and aspirations	No STEM identity, “hated” science, no science aspirations	Ambivalent STEM identity, science aspirations (medicine)
3. Experience of program	Most positive; “Loved everything”	Mixed—enjoyed maths but disliked outdoor, hands‐on, and creative	Most positive	Mixed—found most “boring” but liked parts on Black women STEM professionals
4. Main equitable outcomes				
4.1 STEM capital	Increased	Increased	Increased	No change
4.2 STEM identity work	Some changes	No change	Transformed	No change
4.3 STEM trajectory	No change	No change	Changed to STEM‐related aspiration	No change
4.4 Agency	Increased self‐confidence and eco‐agency	Increased confidence around animals and eco‐agency	Increased self‐confidence	Feeling empowered as Black girl in STEM
5. Practices supporting outcomes	Specific enactments/moments in youth data			
5.1 Equitable pedagogies	“Everyone gets a go” *a*	“There's no such thing as too much sawdust!” *b*	“We were able to say what we wanted” *a*	“Why don't you lead the next session?” *b*
5.2 Broadening what counts as STEM	Hands‐on “doing” and arts *a b*	Hands‐on “doing” and arts *a b*	–	–
5.3 Designing for agency	Eco‐agency *a b*	Eco‐agency *a b*	–	Black women in STEM *a b*
5.4 Loving/caring pedagogy	“Cole is really nice” *a b*	“Do you have a phobia?” *a b*	–	–

Abbreviation: STEM, science, technology, engineering and mathematics.

We then explored the data to identify *enactments of practice* that might have supported these outcomes, guided by the four‐key approaches to equitable practice identified by our literature review, as discussed earlier. We collected both youth‐identified practices, that is, practices that the youth themselves felt had supported them to achieve particular outcomes and researcher‐identified practices, that is, our own theory‐driven data analyses. We adopted a Bourdieusian lens, paying particular attention to how the young people's habitus and capital interacted with the field of the ISL program in question. We were particularly interested in any changes and shifts from dominant norms. The first pass of this process involved looking in detail at each youth's multimodal data to identify any instances of practices identified by young people and particularly those which they attributed to their outcomes. This resulted in the identification of enactments of practice marked in the table with *a*. In the second pass, we worked from each youth's identified outcomes and interrogated their data for any practices mentioned by practitioners and/or in researcher observation notes, from which we identified the enactments in Table 3 marked *b*. These enactments were then considered in relation to the four areas of practice derived from the literature (rows 5.1–5.4), using a theory‐led categorization, moving back and forth between the data and the existing literature. Finally, we worked our interpretations through a Bourdieusian lens, reflecting in particular on the extent to which dominant power relations and doxa were minimally or substantially disrupted. We termed those enactments of practice that seemed to only minimally disrupt dominant power relations, forms of representation and epistemology as “weaker” enactments and we classified “stronger” enactments of practice as those which more significantly or substantially disrupted the dominant field.

Next, we introduce the four case study youth, detailing for each of their ISL program experiences and their main equitable outcomes. We purposively wanted to interrogate the relationship between practices and outcomes from the perspective of the young people in line with the value placed in the critical literature on youth‐centered approaches to analysis and to understanding phenomena from a youth perspective. We then discuss the four key practices that were identified in support of these experiences and outcomes. For each practice, we start by detailing what the practice was and how it was enacted in the settings. We then discuss how and why we judged it as relating to equitable youth outcomes in the data, and finally we offer our interpretation for each, reflecting on the affordances and limits of employing the Bourdieusian lens.

## 3 THE FOUR CASE STUDY YOUTH—EXPERIENCES AND OUTCOMES

### 3.1 Lulabelle (zoo program, positive experience)

#### 3.1.1 About lulabelle

Lulabelle is a white, working‐class girl, who at the time of the research was in receipt of free school meals. She had relatively little science‐related capital within her wider family and was not a regular zoo visitor, explaining that she and her family do not really like or agree with zoos. Lulabelle was interested in science before the program—she also attained well in the subject at school and was in the top set (track) for science. However, her interest and identification with science were mediated by what she perceived as “boring” school teachers and dull, didactic pedagogy (“during school, all we do is stare at a board, write, stare at the board, write”) and when asked by the researchers if she saw herself as a science person, she replied “yes and no,” explaining that although she found science interesting, she also felt excluded by the culture and practices of school science. Her school experiences were marked by being bullied for being into science and “being clever” (“I've been picked on for being the smart one in the class, since I started […], those in my form, they don't really accept me … they pick on me for that”). Lulabelle aspired to be a primary school teacher.

#### 3.1.2 Lulabelle's experiences of the program

Lulabelle engaged enthusiastically throughout the program. She took an active part in all activities and discussions and enjoyed making colorful and thoughtful objects (such as a bird box, bug hotel, ibis feeder) and informative posters (on animal habitats, to inform zoo visitors). Lulabelle frequently offered her views and answers to Cole's questions while always appearing to be mindful of other students. For instance, observation notes record her looking around to check if anyone else wants to speak before answering and giving space to others' views. She particularly loved engaging with the animals (e.g., observing and recording flamingo behaviors; feeding animals) and expressed very positive views about the program both during and after. For instance, when filling in an evaluation form, she asked Cole if she could put “everything” as her answer to the question asking what her favorite thing about the program was. The following exchange, which took place during the program after a day which had included a lot of direct working with animals, also highlights this:

Lulabelle: This was the best day so far!

Evie: Definitely the best day for animals

Lulabelle: I mean like generally, this was the best day I've ever done!

She enthusiastically and extensively completed many homework pages in her portfolio and observation notes record her showing her portfolio book (containing notes, worksheets, photos, and reflections) to her friends, family, and teachers afterward.

#### 3.1.3 Lulabelle's key equitable outcomes

We recorded a range of outcomes from the program for Lulabelle across the outcome categories. For instance, in her follow‐up interview Lulabelle reported gains in STEM knowledge as a result of the program (one of the dimensions of STEM capital), which persisted over time. She also described developing wider dispositions and engaged in more science talk with family members at home, particularly building a shared interest in birds with her grandad, which we interpreted as gains in STEM capital (ii). Although, as detailed in her interviews, Lulabelle had already identified to an extent with science before doing the program due to her competence in the subject at school (“I was already a science‐y person because in my form I'm seen as the one that knows everything”), she felt that the program had made a difference to her science identity work and what sort of science identity she was able to perform, and the ways in which she was recognized for this. In particular, she felt the program had supported her to engage in, and be recognized for, broader forms of science identity work than at school, for instance, through doing hands‐on, practical activities in the zoo and through the creative and artistic tasks. Her experiences on the program also enabled her to perceive new possible futures for herself:

I wasn't very interested [in science] because it wasn't something I could see myself doing when I was older. Then I did the programme and it flipped the switch in a way. I was like, "Oh, I could do this when I'm older" (Lulabelle, follow‐up interview).

Although as she went on to explain, she still maintained her prior aspiration to be a primary teacher. In her follow‐up interview Lulabelle also described some behavioral changes, which we coded as examples of developing critical STEM agency that had followed from her participation, notably more eco‐friendly behaviors:

I've changed my carbon footprint quite a lot…. I used to take the bus to go to [London area] and things like that. Now I walk a lot more because I have my mum to do it with me. To get to places I don't take the car very much, I walk. My recycling, I check what bin I put it in before I put it in the bin.

She also described feeling more able to speak out at school and “develop” socially (“the programme made me realise that I can speak about things and have a laugh about it. So it didn't make me more confident, it just helped me. It just helped develop me in a way”), which we interpreted as supporting agency outcomes.

### 3.2 Star (zoo program, mixed experiences)

#### 3.2.1 About Star

Star identifies as a North African/East European working‐class boy who, like Lulabelle, was in receipt of free school meals at the time of the research. Before participation, Star already liked science, and in interviews described himself (and was seen by others) as “a maths and computing person.” He attended his school STEM club and had taken part in a number of STEM enrichment activities before with his family. Despite attaining highly and being in the top set at school, he described having “problems” with the teaching style of his science teacher.

Like his teaching methods don't really work for me, like, they work for everybody else, but his methods are just copy, copy, copy, check if you know it, copy, copy, copy and just do that again and again and again.

While Star said he did “not really” identify with science, he enjoyed coding at home (“I've learnt from books, on my own”) and proudly listed all the programming languages he knows. He described his family as being “not interested” in programming, although they did support his interests, and had taken him to CERN. Through school, he attended a range of science festivals and events. Star aspired to be a computer programmer.

#### 3.2.2 Star's experiences of the program

Star appeared to be most comfortable during classroom sessions, when performing and gaining recognition for his mathematical and technical knowledge (“I really enjoyed the maths”). Star was frequently loud and tried to dominate group discussion and activities (in relation to both other youth and the facilitator), for example, shouting out and over others, trying to intervene in their projects. In particular, he dominated through displays of intellect and mathematical and scientific terminology and knowledge, which we read as performances of *talking science through muscular intellect* which involve “confident, often ‘arrogant,’ competitive displays of using ‘correct’ scientific terminology, often in the context of verbal ‘one‐up‐manship’ and as part of bids for public voice and attention within the classroom” (Archer et al., 2018), through which students assert scientific and social dominance. Star found the perceived lower mathematical abilities of his peers on the program annoying (“It was really frustrating cause no one could do the maths right!”) and repeatedly corrected (and berated) other youth if they got facts “wrong,” mispronounced words, and so on. He disliked getting wet, muddy, and dirty, found the animals “scary” and disliked art/craft activities and teamwork (“it was fun but there were some parts I didn't like… the arts and falling into the mud. I found the teamwork sections quite frustrating”) and would often play up during the parts he did not like, exhibiting challenging and disruptive behavior on a number of occasions. Despite being vocal about not enjoying these aspects, Star was observed on some occasions voluntarily continuing with particular activities (e.g., staying on at the end of an activity to plant herbs with Cole; suggesting technical improvements to a bug hotel tower). Over the week, he also managed to overcome some of his fear and anxiety about animals and successfully completed most animal‐related tasks.

#### 3.2.3 Star's key equitable outcomes

Star recorded increased STEM knowledge (“I learnt about the different species of animals and I think animal taxonomy or something”). While he already expressed very pro‐STEM dispositions at the start of the program, some further changes in disposition were noted, which we interpreted as evidence of some increases in STEM capital. For instance, despite initially saying that he “hated” biology, he showed interest and curiosity over the program (e.g., on Day 3, he was recorded asking “if there were no more flamingos, would shrimp still exist?”). Star's key equitable outcomes related to what we interpreted as examples of *eco‐agency*. For instance, he felt that he had learned how to take care of animals better and how to look after the environment. In the follow‐up session, he described how he now takes better care of his cats and expressed a new commitment “not to wreck the environment.” He had also given up meat (“I went pescatarian.”) and had become more confident around animals.

### 3.3 Crystal (STEM club, positive experience)

#### 3.3.1 About Crystal

Crystal is a white, working‐class British/Irish girl. She is quiet and softly spoken (describing herself as “quiet and shy”). Before the program, she was not a regular ISL participant, although she had previously visited some settings with family members (e.g., she described once being taken to an ISL holiday drop‐in session at a local STEM organization). Before the program, she did not espouse any sort of science identity—indeed, she said she “absolutely hated” science and technology (“sometimes I find it difficult to understand”) and found school science “hard” and “boring.” She aspired to be a lawyer. She was persuaded to join the club by her mother (“My mum kind of persuaded me, saying it would be good for me, especially to boost up my confidence and then I just decided to come and I enjoyed it”).

#### 3.3.2 Crystal's experiences of the program

Crystal was a quiet but enthusiastic participant in the girls' STEM club. She did not often offer her views publically, but preferred to talk softly and quietly either one to one or in small groups. She was often recorded smiling and always engaged thoughtfully and fully in club tasks and activities. She stood out among her peers by doing by far the most homework between sessions, bringing in photos, videos, writing, and drawings that she had made during her own time between sessions for her portfolio. She enjoyed the club sessions and coding days, which she found “cool.” She attended both of the day visits and seemed to really enjoy her involvement in the program.

#### 3.3.3 Crystal's key equitable outcomes

Crystal derived a range of positive outcomes from participating in the program. She felt she developed “more understanding” of STEM (particularly science and technology) as a result of her participation, although she found it hard to articulate exactly what she had learned. One of her most noticeable equitable outcomes was a change in science dispositions (“I used to absolutely hate Science and now I like it”), a view which her friends on the program also concurred with; her best friend Tori told us “What I'm saying is, like, 'cos Crystal used to hate Science, but then, 'cos she got into, like … she came in, she had more fun.”. Crystal also described having broadened her understanding of STEM careers and said that she regularly talked to her mum about the program and what she had done and learned (which we interpreted as STEM capital (ii)). Crystal's personal sense of her STEM identity shifted as a result of participating (“I would say I'm a little bit of a science person”) and also felt that others recognized her more now as a STEM person. Crystal's aspirations also changed, from wanting to be a lawyer at the start of the program, to wanting to be an architect (when followed up 6 months later), which she attributed to having developed more interest, knowledge, and confidence in STEM through the program (she agreed with the statement “the programme has made me want to do more STEM in the future,” something she said she had not considered previously). Finally, Crystal also considerably developed in her personal confidence over the course of her participation.

### 3.4 Innocent (STEM club, mixed experiences)

#### 3.4.1 About Innocent

Innocent identifies as a Black British African, lower middle‐class girl. She took part in the girls' STEM club program. She did not regularly participate in ISL previously but was already interested in science before taking part. Innocent had a long‐standing aspiration to be a doctor (either in cardiology or orthopedics). Neither parent has a science background although a couple of her extended relatives (overseas and in London) worked in medicine. She had science kits at home and watched various science‐related TV programs. At school, Innocent liked biology and chemistry but “hated” physics. She was very academic (“I like revising, I actually like revising”) and had lots of tutoring outside school (in a range of subjects) to support her attainment. She and her best friend, Dani (who also took part in the program) were both very focused on academic success and often competitively compare grades with one another. Innocent performed a cool (fashionable) Black femininity and did not interact much with other girls on the program, apart from her best friend, Dani. Although liking science and aspiring to be a doctor, Innocent expressed an ambivalent science identity, feeling that she is not recognized by others as science‐y (“cos no one really asks and no one really cares”).

#### 3.4.2 Innocent's experiences of the program

Innocent had mixed experiences. She mostly found the weekly sessions “boring” in terms of their science content (much of which she felt she already knew) and format (she found many of the activities unengaging and insufficiently experimental—she wanted to do science practicals using “real” apparatus and equipment). Her boredom was reflected in both her interview comments during and after the program (“I'm not gonna lie, it's boring”) and in observations, when on a number of occasions field notes record Innocent as talking or laughing with her best friend and not doing the set tasks. Innocent recorded mixed experience, enjoying some parts of the program (where it gave her new knowledge or skills) but disliking others, which she felt she already knew, had covered at school previously, or were dull or lacking in value (“all boring… I wanna be learning something new”; “so it was really like, we'd already done that at school, so I don't want to do it out of school”). Innocent was most engaged when introduced to new concepts and content that she felt were helpful for supporting and enhancing her academic attainment—when she encountered new things that she had not come across before, both in terms of STEM content but also black women in STEM (which we would interpret as in line with Bourdieu's proposal that individuals are driven by a desire for accrual of symbolic capital). Conversely, she disengaged from content and aspects of the program that she felt she already knew and/or were “basic.” Like Star, Innocent was observed on a number of occasions resisting or subverting opportunities for learning that she felt were boring or repetitive of something that she had previously covered in school. She did not like the hands‐on making sessions in the club, as she felt these lacked sufficient “action”—for instance, she wanted more actual experiments and equipment and more new content learning.

#### 3.4.3 Innocent's key equitable outcomes

Innocent felt that the program made little change to her knowledge, dispositions, and identity as she was already interested in science and felt that she was “good at” the subject, although she also was ambivalent as to whether she saw herself and was recognized by others as being science‐y. Innocent did feel that participation in the program had supported her science recognition by others, and said that she felt her parents and teachers now realized how serious she is about her medical science career aspirations. She also felt that the program had reinforced her prior medical aspiration (“It's made me realise that this *is* what I want to do when I'm older”), which she attributed to the focus on Black women in STEM. The program, she said, had emphasized to her that she would stand out, which she saw as a potential mark of distinction (“I would want to be part of a career where there aren't many people like me because I would stand out”). The most notable equitable outcome was that Innocent felt that participating in the program had empowered her, particularly in terms of Black women in STEM, whose presence and contributions had been invisible and ignored within mainstream school STEM education (“So I didn't know about people that are in science. I've done maths and stuff like that but then there aren't many women in it”).

## 4 PRACTICES SUPPORTING EQUITABLE YOUTH OUTCOMES

### 4.1 Centering youth: Framing ISL through the identities, values, and experiences of youth

As discussed below, we found some examples of weaker and stronger enactments of the practice of Centering Youth as relating to the four young people's experiences and outcomes. We also identified some gaps and missed opportunities. Notably, we did not find many examples of youth being clearly supported to be producers of STEM. In addition, while (as below) we identified examples of the programs connecting with young people's interests and experiences, these tended to be at a more superficial level and were not leveraged toward more consequential learning that matters, such as learning that involves the challenging of societal inequalities.

With respect to weaker enactments, both programs sought to appeal to young people's interests, using topics, activities, and examples that were generally reflective of youth interests. In line with our conceptual framework, we interpreted such instances as weaker enactments because they did not substantially rework relations within the field in ways that disrupt dominant power relations, For instance, Madison (the practitioner) chose and controlled the popular commercial music that was played during the girls STEM club sessions and the girls were not invited to suggest or choose songs to be played that might reflect their own interests and taste. Both Innocent and Crystal commented that they liked the relaxed ambience created by the music, but it did not seem to impact in any consequential way on their outcomes.

We identified stronger enactments as those who engaged more meaningfully with the participating youth, for instance in more participatory ways, to tailor and shift the content and format of the sessions and activities to their own interests and needs and in ways that recognize socio‐historical injustices. For instance, our field notes record repeated examples of Cole verbally celebrated all students' contributions, valuing the diverse cultural knowledge, experiences, and identities that they brought with them, and recognizing and foregrounding their existing knowledge (e.g., relating to habitats, eco‐systems) from different cultures and religions that youth shared during the sessions. We interpreted these practices as supporting Lulabelle's equitable outcomes and her valuing of the program as connecting with her own interests and “not like school.”

Stronger enactments involved more fundamental changes to dominant power relations, for instance, as exemplified by a moment, captured by our observations (and discussed afterward in an interview with Bobbi), during a club session when Bobbi noticed that Innocent did not appear to be taking part. Following a short discussion between the two, Bobbi asked if Innocent might be prepared to lead the next club session, disrupting the usual practice of an adult facilitator leading and steering the content and learning. Innocent did so the following week with competence and authority, as recorded in the observation notes:

Innocent stands at the front and confidently introduces the session topic on space and Black women who have worked in space science and computing. She reads out a short introductory extract from the handout for today's session. Madison plays an extended YouTube clip about the film *Hidden Figures* (about Katherine Johnson). One of the Black young women exclaims “Oh, she's Black!” when she realises that Katherine Johnson is Black. Innocent replies “Yeah, haven't you heard of the film?” When the phrase ‘coloured computers’ is mentioned in the film, Innocent raises her eyebrows at the other girls. The girls watch attentively, occasionally exclaiming “woah”. At the end of the clip, Innocent turns to the girls and says “Wow! Any thoughts about the film?” She invites discussion and contributions, giving each student space to share their views. The girls ask questions, such as “can you die in space?” “What happens to a dead body in space?” Innocent tells them “you can apparently drown in space” which she explains is due to droplets of exhaled water condensing in a space helmet. There seems to be a greater amount of student talk about science than we have previously observed in any of the club sessions. The two (white) staff observers (one teacher, one teaching assistant) who sat at the back of the class start to interject and answer the questions. They take over the conversation and one of them starts to tell a long anecdote about what computers were like when she was at school.

We interpreted Bobbi's inviting of Innocent to lead the session as attempting to shift adult–student power relations toward centering and supporting youth agency—although this remained a one‐off example and was not worked through the program subsequently. We observed that Innocent participated more vocally and actively in this session compared to other sessions and that, as a group, the young women raised questions and shared ideas more in this moment compared to other club sessions, which may have been facilitated by the shift toward more youth‐centric pedagogy. However, we also observed that the two white science teacher observers, who were required by the school to be in the room for safeguarding reasons, but who normally sat quietly at the back of the room and rarely participated, became more vocal in this case and attempted to assert themselves and control the discussion when it was led by Innocent. This happened despite the young women's talk and curiosity questions being ostensibly relevant to the space theme of the session. We suggest that the staff interjections might be interpreted as perhaps an unconscious response to the shift away from the more hierarchical adult–student relations that are common in science lessons toward more youth‐centric pedagogy.

#### 4.1.1 How this practice related to equitable youth outcomes

Lulabelle and Crystal explicitly linked enactments of what we identified as enactments of the practice of centering youth, to their own positive outcomes. For instance, during her follow‐up interview, Lulabelle attributed her gains in knowledge and confidence to Cole's practice, which she contrasted with her own disengaging experiences of school science. Likewise, in a follow‐up interview, Crystal directly attributed her experience in the club of being able to “say what we wanted” to her increased enjoyment and understanding of STEM. As she explained:

Before I hated programming and it just really confused me but now I understand it a lot more and I enjoy it a lot more. It's not as boring as it seems. I used to absolutely hate science and now I like it […] When I first started the class, I wasn't too sure about it, and then after the first lesson I realised I really enjoyed it and so I enjoyed it more, which made me more confident.

When the researcher probed what it was about the sessions that had enabled this, Crystal replied “how we were able to say what we wanted.” That is, she liked that the young women were given the opportunity to choose which STEM activities they wanted to do in each session. We interpreted this as an example of youth‐centered practice in that it represents participatory and youth‐led principles.

Innocent and Star did not explicitly articulate a link between their experiences of youth‐centered practice and their equitable outcomes, but we observed that Innocent appeared much more engaged when she led the space session and that the other girls in the group engaged in the greatest volume, and highest quality of talking science (Lemke, 1990) during the session that Innocent led, compared with all the other facilitator‐led sessions—albeit until the two science teachers intervened. However, we suggest that the hijacking and closing down of this moment by the two white staff members may have played a part in constraining Innocent's outcomes and may have been part of the reason that she expressed ambivalent feelings about the program in her interviews during and after the project.

### 4.2 Interpretation

The two programs could usefully have done more to support young people to be producers of STEM and to use STEM more meaningfully to engage with consequential issues that impacted their lives—although we did identify some examples of practices that sought to move in this direction. We interpreted the equitable potential of the stronger enactments of practice that were identified as being predicated upon: (i) helping to reduce the distance between youth habitus/capital and the field of STEM and ISL, as Bourdieu suggests that success in a given context is predicated on a close fit between habitus, capital, and field and (ii) reworking dominant power relations between adults and youth within the subfield of the program. In particular, we interpret the equitable potential of weaker enactments of inclusive pedagogy as constrained because they do not rework dominant norms and power relations—even though the programs and content may be generally orientated toward youth in ways that some participants may enjoy or appreciate. However, the equitable potential of practice will be constrained when doxa is left in place, precluding more consequential changes in power relations. Hence, we identified stronger enactments as those who helped to support nondominant youth to have power and authority in the sessions and might be seen as examples of purposive working toward more symmetrical relations of expertize between teachers and learners within a particular setting (Lave & Wenger, 1991) and challenging of doxa. Although, as noted in Innocent's example, these moments were relatively rare and were resisted by those who are used to having power and voice within STEM learning settings.

### 4.3 Challenging elite STEM practices, epistemologies, and representations

We identified some weaker and stronger enactments of this practice and also some missed opportunities. In particular, both programs had a prescribed curriculum that had been set in advance by the practitioners and which left little room or opportunity for youth ownership and direction. While, as discussed below, we found examples in both programs in which normative representations of STEM were challenged (for instance through the showcasing of Black women STEM professionals) and some attempts to broaden traditional STEM epistemological approaches (for instance through the hybrid creative STEM activities in the zoo program); on the whole there were few examples of youth being supported to challenge normative STEM epistemologies and representations or to rework dominant elite STEM cultural discourses and practices. However, we did find examples in both programs of attempts to present and enable youth to participate in STEM in ways that were more inclusive than prototypical STEM, a point that young people acknowledged and felt had supported their participation and outcomes.

We observed a spectrum of enactments of challenging elite STEM practices, from weaker to stronger versions, with the more equitably consequential (stronger) enactments being predicated on a disruption of dominant STEM educational norms, rather than an uncritical inclusion of youth into normative STEM. An example of a weaker enactment comes from the STEM club, where we observed that Madison noticed that Crystal was very quiet and shy and tended not to speak during whole group discussions, so tried to find ways to speak with her individually and would explicitly invite and make space for Crystal's input during whole group work. These enactments focused on providing interpersonal support to an individual student, which was valued and welcomed by Crystal. However, the enactment did not seek to change the underlying norms, structures, or the epistemological basis of the sessions. We also interpreted weaker enactments as those who sought to broaden what counts as STEM through the use of hands‐on and fun activities but which did not reformulate dominant STEM epistemologies. For instance, our observations recorded that in one of the club sessions, the girls were presented with a recipe style activity with instructions for building and flying paper airplanes (to exemplify learning around aerodynamics). While many of the girls enjoyed the activity, as Innocent was both observed to comment on and as she reiterated in a follow‐up interview afterwards, the activity directly replicated a task that she had previously done in school science and the outcomes were already known, which she found “boring” and felt did not constitute a “proper” (authentic) science experiment (e.g., Chapman et al., 2017). We interpreted the club's practice of fostering of a youthful, informal party atmosphere, by providing food, games, and playing pop music in the background of all sessions, as helping to challenge notions of STEM as serious, adult, and academic. However, as discussed above, we interpreted these as also relatively weak enactments in that they did not extend to letting girls decide, for instance, what was music was played.

In the zoo program we identified some stronger enactments of this practice as exemplified by observations of (i) youth engaging in hybrid arts/craft–science activities and undertaking authentic zoo work (e.g., feeding animals, building enrichment objects for enclosures, planting a sensory garden) and (ii) Cole's practice of challenging some young people's dominant ways of speaking science through muscular intellect to value and support broader forms of engagement. With respect to the former, while in one respect, both the zoo and the club programs used hands‐on activities and hybrid arts/craft and STEM activities, we interpreted the zoo's approach as having greater equitable potential because it was less formulaic, more aligned with authentic zoo tasks, and was more clearly orientated to challenging dominant STEM epistemologies. For instance, we observed that Cole explicitly set up and supported group values and an ethos that entailed both mutual respect but also challenged dominant ways of being and doing in science, such as the practice of talking science through muscular intellect. This can be seen in the following observation notes where Cole gently, but firmly, closed down Star's attempts to dominate an activity in which the young people are making enrichment feeding tubes for an Ibis enclosure:

Star is telling other youth off about putting too much sawdust in their tubes. He pulls another youth's tube out and empties it—they get annoyed. Cole says loudly “there's no such thing as too much sawdust!”

Cole asks Magic (a very quiet Black African boy) to make a list to record the size of people's bird boxes. Star repeatedly shouts out “I can do it!” Cole says “no, Magic will do it”. Star repeatedly tries to take over, but Cole keeps stressing that Magic can do it, and encourages Magic, who completes the task.

We did not record any specific equitable outcomes relating to Star and this practice, however we note that in both interviews and observations, Star did not mention or complain about his experiences of his behavior being managed in this way and still recorded gains in STEM capital and other outcomes. Nor was there any discernible detrimental impact on his STEM identity work or STEM aspirations.

As Lulabelle explained in an interview, Cole's careful attention to, and disruption of dominant power dynamics meant that Other students were able to have a voice in the sessions, “everyone gets a go”:

Cole is really nice, like, he engages everyone, he makes sure, like, everyone's engaged, where some of my teachers, they all like pick, like, the smart kids really … they pick favourites, but I think with Cole, he like equals it out, like, really well, so everyone gets a go.

In the club we observed a stronger enactment of this practice as being the foregrounding of Black women STEM professionals, which reformulated dominant notions of STEM as largely practised by white men—which is discussed further below in relation to the practice of supporting young people's critical STEM agency.

#### 4.3.1 Relationship of the practice to equitable youth outcomes

Lulabelle felt that the authentic tasks that they undertook on the zoo program ‐ such as caring for and working with the animals, and building enrichment objects for enclosures ‐ were an important and valuable way of learning and engaging with science. As she explained in an interview:

Because you get to, you learn, in my opinion, you learn so much more because you're actually *doing* something, so you get to go out and be with the animals and you get to learn that, you get to learn about their diets and everything like that and I think that's really cool.

We interpreted Lulabelle as signaling a breaking down of the traditional hierarchy between elite academic science learning and the learning through doing that she had experienced on the program. Likewise, Crystal talked in an interview about the informal atmosphere of the club and the lack of pressure to “get the answer right” helped her feel more relaxed and confident, which we interpreted as supportive of her STEM identity work.

While enactments of the practice of challenging elite forms of STEM seemed to support Lulabelle's and Crystal's enjoyment and equitable outcomes, it was not enjoyed by Star, who was vocal about his dislike of the animal‐related, creative, and outdoors “messy” activities on the zoo program. However, our observations and interviews with Star suggested that his discomfort with these aspects did not constrain his achievement of equitable outcomes. Indeed, we would attribute one of Star's consequential outcomes—namely becoming less fearful of animals—as resulting from his engagement with animals as part of the program. That is, even though he was adamant in the interviews and observations that he did not enjoy these aspects, they still seemed to support equitable outcomes for Star. Another example is provided in the following observation notes, which show how Cole's practice of challenging elite STEM practices and representations seemed to relate to Star's equitable outcomes. The notes are from a session in which young people chose and planted herbs and sensory plants in a large new raised bed in a central part of the zoo:

The group get ready to go outside, Star complains that he dislikes outdoor activities, saying “I hate getting my hands dirty”. The young people stand around the new raised bed and Cole explains the importance of zoo spaces being inclusive and accessible to all. Cole asks them to think about what they can do to ensure that everyone can enjoy these public spaces, e.g. what could they do for people who cannot see or who cannot read the language on the signs? The young people and Cole talk about the five senses and what different smells and touch they like and how they feel when they smell, see or touch something they like. They consider the importance of sensory spaces to people with learning impairments and how the height of the bed should make the plants more accessible to wheelchair users. Cole shows them a store of plants and invites them to choose plants they think would work well and plant them wherever they like in the bed. Most youth start enthusiastically choosing and planting. Star seems more reticent than the other young people and asks Cole questions about where he should plant the herbs he has selected. He starts planting later than the other young people and keeps seeking reassurance from Cole that he is doing it “right”. A researcher asks Star if he had done this before and Star talks about his garden at home and what vegetables his family grow. He seems to relax and starts planting, saying that he is putting his plants at the edge of the bed so people can touch and feel them. The other young people finish and go off to play on the playground that is nearby. Star stays behind saying “but there are more plants that need planting”, he continues chatting with Cole as he plants more plants.

We interpret Cole's framing of the activity, in which youth participate in an authentic zoo task that is framed in terms of social inclusion, as challenging dominant STEM epistemologies and supporting more equitable forms of engagement. We observed that Star engaged in more thoughtful and co‐operative behaviors during this activity that stood in stark contrast to his performances of talking science through muscular intellect that we commonly observed during more traditional classroom sessions.

#### 4.3.2 Interpretation

Criticisms have been made of the mainstream ISL field for predominantly reducing issues of inclusion to the question of how to enable more diverse communities to access STEM learning (e.g., Dawson, 2014) and how to assimilate diverse communities into STEM (Bell et al., 2009), rather than seeking to transform what STEM is and how it is practised, to better meet the needs and interests, and recognize the identities and expertize, of diverse participants (e.g., Hernandez et al., 2013). We interpreted the equitable potential of this practice as predicated on the extent to which it countered the doxa of school science, notably in these examples, the alignment of science with abstract and academic book learning, whiteness and masculinity, hierarchical relations between an authoritative/expert teacher and a novice student and, to a lesser extent, seriousness and formality. That is, equitable outcomes will be more strongly supported where enactments of the practice sought to reconfigure dominant power relations within the subfield. This aligns with the wider literature on social justice approaches to science education, which argue for the value and importance of broadening what counts as science and who can be recognized as being good at STEM (e.g., Calabrese Barton & Tan, 2010; Calabrese Barton et al., 2008)

Thus, we suggest that the equitable potential of enactments of the practice of challenging elite STEM practices in the programs depends upon the extent to which they rework dominant power relations (between educators and youth and between youth) and challenge dominant STEM doxa—namely STEM epistemology and dominantly celebrated performances of STEM, such as talking science through muscular intellect. While both programs only partially achieved these aspects, we suggest that our analysis helps to highlight ways that practice might be usefully further developed in future—a point that we return to in conclusion.

From a Bourdieusian perspective, we suggest that stronger enactments would also redress the alignment of science with middle‐classness, given that abstract and academic knowledge is aligned with middle‐class ways of knowing, which are traditionally accorded higher status than vocational and experiential ways of knowing. Such constructions are underpinned by a Cartesian dualism in which the separation of mind/body and science/art are also aligned with respective binaries of masculinity/femininity and whiteness/Otherness (e.g., Francis, 2000). Hence, the practical making and creative/artistic activities observed in the zoo can be understood as trying to close the gap between the field and nondominant habitus, enabling related nondominant youth capital to be valued and leveraged—an approach that is widely discussed and enacted within funds of knowledge approaches (e.g., Moll et al., 1992). As Bourdieu argues: “it is one and the same thing to determine what the field is, where its limits lie, etc., and to determine what species of capital are active in it, within what limits, and so on” (Bourdieu & Wacquant, 1992, pp. 98–99). In this way, we argue that it is not the nature of a task or activity (e.g., as being hands‐on or creative) per se that supports equitable outcomes. Rather, the equitable potential of this practice relates to the extent to which dominant doxa and power relations are shifted, or not. We thus suggest that the potential for achieving equitable outcomes among under‐served youth will be enhanced where programs actively disrupt and challenge the wider doxa of prototypical school science, broadening dominant ways of doing science and being recognized.

We interpreted the example of Star overcoming his fear of animals and “getting dirty” as achieved through experiencing, and overcoming, a disjuncture between his habitus and the field. Bourdieu proposed that a transformed habitus can arise when disjunctures between the habitus and field are resolved through the development of new personal reflexivity (see also Reay et al., 2009; Yang, 2014). We would further speculate that, in the case of Star, it may not be solely the experience of disjuncture per se that generates Star's new disposition toward animals or getting dirty, but that the productive potential of the experience is enhanced and mediated by the fourth identified practice (discussed below), namely respecting and valuing young people's identities in STEM through loving/caring pedagogy.

### 4.4 Supporting young people's critical STEM agency

Examples of the third practice, supporting young people's critical STEM agency, was observed to some extent in both programs. However, we did not find examples of young people being supported to exercise their voices or enact critical STEM agency in relation to issues that they personally cared about beyond the context of the programs, apart from, as discussed next, changes in young people's environmental agency in the zoo program. We observed that the zoo program had been intentionally designed to support youth STEM agency in relation to the environment (eco‐agency). For instance, youth were actively supported to develop environmental awareness and critically reflect on and take action in their lives to protect the environment through, for instance, recycling, protecting habitats, and thinking about their carbon footprint. In the girls STEM club, the program was specifically designed to support young women's critical awareness of gender and racial inequalities in STEM participation and involved the showcasing of pioneering Black, White, and Middle Eastern women who had overcome these inequalities to achieve a career in STEM (as exemplified by the earlier example of Katherine Johnson and the Hidden Figures film).

In both programs, we found examples of this practice threaded across different sessions, rather than being a one‐off or standalone feature, which we suggest helped strengthen the impact of the practice. For instance, in the zoo, the themes of environmental awareness, action, and sustainability were recorded in all the observed sessions and during many informal moments too, during downtime in lunch breaks and at the end of the day, when Cole chatted with the young people. In the girls' STEM club, each session began with a focus on a particular woman in STEM, with a printed sheet, photo biography, and a short film clip about them. The two external, off‐site visits that the girls took part in (a weekend coding day and a school coding day at a city tech company), as well as the club ethos were also explicitly framed in terms of challenging women's underrepresentation, introducing them to women STEM professionals. For instance, the two off‐site visits included women STEM professionals talking to the girls about their lives and careers and how they had overcome gendered barriers to progression. However, we did not find examples of the young people being supported to extend their agency and advocacy more widely in relation to these issues through the programs.

#### 4.4.1 How this practice related to equitable youth outcomes

As detailed in the individual case studies, in their interviews, three of the youth (Innocent, Lulabelle, and Star) identified this practice as important and recorded some related equitable outcomes. For instance, in her follow‐up interview, Innocent described feeling an increased sense of agency as a young Black woman in STEM as a result of the weekly sessions profiling women in STEM, with the two sessions that had focused on Black/minority ethnic women being particularly influential. As Innocent put it “that was good to see because it's usually predominantly a white career.” She continued:

Yeah I liked it cos it gives you something about, cos you wouldn't usually listen about female mathematicians, it's usually men, especially in the western world. So knowing that women can do it as well is really empowering to little girls like us, it makes me feel better.

As detailed in their case study overviews earlier, in their follow‐up interviews, Star and Lulabelle both also reported significant changes to their environmental agency, having developed new sustainable habits, such as recycling, and in Star's case, turning pescatarian.

We did not notice any specific equitable outcomes related to this practice for Crystal—although she reported increased personal confidence and interest in STEM as a result of the program, we did not have any evidence that these related specifically to the foregrounding of women in STEM.

#### 4.4.2 Interpretation

As discussed in relation to the first two practices, we interpreted the potential consequentiality of this practice as grounded in its disruption of dominant power relations, specifically, the ways in which enactments challenge the doxa of STEM as white and male and positioning youth as having the agency to make a difference to the planet. While the observed outcomes on the two programs were modest, we interpret the effectiveness of enactments as being enhanced when embedded—that is, equitable outcomes appear stronger when enactments are repeated and sustained across a program. From a Bourdieusian perspective, this embedded repetition is important for enabling cultivation of habitus and enabling the practice to be a core feature of the field.

Bourdieu challenges the equitable potential of critical consciousness work, arguing that it is akin to a false consciousness in that it hides the continued dominant workings of power. However, we disagree and propose that the practice represents a useful disruption of dominant relations in the field, which can help support equitable youth outcomes and hence can be interpreted as valuable and consequential. As with the previous practice, we also interpreted this practice as being amplified and mediated by the fourth identified practice, namely respecting and valuing young people's identities in STEM through loving/caring pedagogy.

### 4.5 Respecting and valuing young people's identities in STEM (through loving/caring pedagogy)

We found examples of enactment of this practice but also some gaps. For instance, we found very few examples in the STEM club program of young people's lived experiences and community wisdom being leveraged and valued. As previously discussed, the participatory nature of the programs were also constrained by the prescribed curriculum and lack of opportunities for young people to take ownership of the content and knowledge. However, we identified examples in both programs of young people being recognized as scientific. We also noted a particular feature of Cole's pedagogy with youth on the zoo program that we felt supported and enacted this practice, namely his caring pedagogy, which we see as an example of Black and feminist critical literature has termed the pedagogy of radical love. The interviews and observation data suggested that Cole's relationship with the young people was characterized by mutual trust and liking between himself and the youth, which extended beyond the bounds of formal sessions and were evidenced through naturalistic, informal, friendly conversations during breaktimes or at the end of the day. We observed that these enactments involved actions and expressions of care by the facilitator toward the young person (e.g., valuing and engaging with them about their lives, sharing hopes and fears) and explicit foregrounding and recognition of societal injustices (e.g., racism, sexism) within his pedagogy. Cole's pedagogy also involved a central valuing of young people's identities, interests, and existing knowledge within the program.

For instance, in the interviews, Lulabelle and Star highlighted how they felt respected and valued by Cole and underlined his care toward them. Observations recorded examples of open, trusting, and reciprocal talk between youth and Cole. For instance, Star repeatedly asked Cole questions on a wide variety of topics, during and outside the sessions. These discussions often involved mutual trusting disclosure, such as a conversation we observed when Star asked Cole if he had a phobia, which segued into a personally meaningful discussion between the two about their respective fears and how they tackled them.

We did not find this practice to the same extent in the club program, although in their interviews and follow‐up interviews youth described the regular facilitator, Madison as “nice,” “friendly,” and fun. However, they did not appear to build close relationships, a point that Madison also recognized and reflected on afterward in an interview:

What would I do differently? I think I'd spend a bit more time getting to know them, I think that's something that I kind of lacked with, it was just, I don't know, just having a sit down, getting to know what they like, what they don't want to do.

We interpreted the interaction between Bobbi and Innocent (described earlier) as closer to a more meaningful enactment of this practice, although it was only observed on one occasion. As Bobbi later explained in an interview with us, she initiated her intervention based on a feeling of connection with Innocent (“I recognised what she was feeling”), as a young Black woman from a similar socioeconomic background who was feeling alienated and disenfranchised by the dominant norms of the session at that point. Hence we would interpret Bobbi's interaction as driven by a politics of love.

#### 4.5.1 How the practice related to equitable youth outcomes

The young people's relationship with Cole was interpreted by youth and researchers alike as important and consequential for supporting equitable youth outcomes. For instance, Lulabelle talked at length in interviews about how important Cole's practice was to her enjoyment of the program and production of equitable outcomes. We interpreted the caring and mutually respectful relationship that we observed between Cole and Lulabelle as being instrumental to enabling her to have a voice, engage, and to be both recognized and respected for her identity work on the program. Likewise, Cole's relationship with Star was critical to the young man's development of eco‐agency, one example of which we traced back to an informal, after‐class conversation when Star asked Cole, “Can I ask you why you are vegetarian?” The two went on to have a discussion and field notes record Star as appearing deep in thought afterward. Five months later, when he was re‐interviewed at school, Star explained how, following the program, “I went pescatarian.”

#### 4.5.2 Interpretation

From a Bourdieusian perspective, Cole's strong enactment respecting and valuing youth through a practice of loving/caring pedagogy was instrumental in setting both the emotional tone and the rules of the game for the zoo program, based on an ethics of mutual respect and care. We interpret Cole's practice as seeking to reduce power inequalities between himself and the youth and among the youth—however, we struggled to find ways in which the Bourdieusian lens added any further interpretive power to the findings. This is perhaps due in no small part to Bourdieu's interpretation of affect and emotion as negative and constraining forces, leading to the reproduction, rather than disruption or transformation, of the habitus—a position that has attracted strong critique (e.g., Probyn, 2004; Wetherell, 2012). Feminist extensions of Bourdieu have also predominantly focused on the role of negative emotions in the reproduction of classed inequalities, such as fear and disgust (as expressed by the middle‐classes toward the working classes and/or working class shame, anger, and envy, e.g., see Lawler & 1999, Skeggs, 2004)—but there has been relatively little sociology of education work on love (cf. wider sociological work on family and/or romantic and intimate relationships, e.g., Carter, 2015).

In contrast, we found that wider critical educational work—but particularly Black/feminist theory—offered a much more productive lens for interpretation of this practice, notably research that has drawn attention to the importance of a pedagogy of love. Freire (1970) argued that a politics of love can enable a breaking down of the hierarchy between teacher and learner, something that we recognized as a key feature of Cole's practice. In her powerful essay *Love as the Practice of Freedom*, bel hooks (1994) traces the presence and power of love as an organizing principle from the civil rights movement through to contemporary movements and struggles. She argues that love is central to liberation efforts—key to creating the conditions for dialogue and to overcome the culture and ethics of domination. Hooks argues that love goes beyond self‐interest, helps span intersectional differences and directly challenges dominant cultural norms of self‐interest and violence, the latter being both the mechanisms and basis of exploitation and domination. She also argues how love enables educators to recognize the hurt and oppressions suffered by those who are dominated and helps provide a collective, community‐based orientation for action. Importantly, “A love ethic emphasizes the importance of service to others” (hooks, 1994) which strengthens compassion and deepens insight—avoiding objectification and deficit views of the Other—all key principles underpinning socially just educational approaches. These principles resonated with our interpretations of Cole's pedagogy, and through his articulation of his commitment to service in support of minoritized youth and his recognition of societal oppressions and injustices within his practice with the young people.

As explicated by Nasir et al.'s (2019) educational work with Black young men, love can be understood as both a productive resource and a political statement in “culturally responsive, caring and loving pedagogies” (p. viii). The authors powerfully argue that “society and the schools embedded within it have made love a foreign thing for Black males.” Indeed, they explain how “The American school, with few exceptions, is too often the place where Black students come to know that they are despised, feared and deemed to be of little to no human value in the world.” In response, an emancipatory pedagogy involves the centering of care and love, reflecting a pedagogical commitment to softening the power differential between students and teachers in support of greater relational equity in the space (DiGiacomo & Gutiérrez, 2016).

We thus interpreted Cole's pedagogy—and the respect, love, and care he exhibited toward the youth on the program—as enactments of equitable practice that was strongly orientated to social justice values. Cole's embodied identity, as a gay, mixed‐race man from a working‐class background, who is also registered as disabled, may also have helped him to identify with and feel a sense of community with the young people, but we primarily interpreted his values and practice of a pedagogy of love, as being particularly powerful in supporting the young people toward equitable outcomes.

## 5 DISCUSSION

In this paper, we have sought to contribute to thinking within both ISL and the sociology of education. We applied a Bourdieusian analytic lens to multimodal qualitative data from two London (UK) informal STEM education settings (a local authority run zoo and a girls' STEM club), focusing on four case study youth, aged 11‐14, from communities traditionally underrepresented in STEM. Through our analysis, we sought to advance understanding of what and how particular enactments of practices might have supported the achievement of consequential outcomes for participating youth—as well as identifying where and how the programs might have supported young people's equitable outcomes more strongly.

We explored enactments of four practices that were identified from the wider critical literature: centering youth; challenging elite STEM practices; supporting critical STEM agency; respecting and valuing young people's identities in STEM. We identified how some aspects of these practices were enacted across the two programs through the perspectives of four focal youth, identifying the extent to which these enactments helped to support equitable youth outcomes. We also noted where key aspects of the practices were not evident, which we suggest may have constrained the achievement of equitable outcomes among participating youth. Using our Bourdieusian lens, we differentiated between weaker and stronger enactments of practice and suggested that stronger enactments of practice are underpinned by a shifting of dominant power relations and a challenging of doxa, that is, dominant epistemic, discursive and representational aspects of science which determine what and who gets recognized as being scientific and how STEM is practised and experienced. We found that stronger enactments of practice related to more consequential and longer‐lasting equitable outcomes for youth—although we do not claim that the youth in question experienced significant transformations to their wider lives and there were many ways in which the programs could have enacted these more strongly and consistently. Rather, we use our analysis to help articulate the conditions under which enactments of the four areas of practice might be most beneficial to supporting equitable youth outcomes.

We extrapolate that the equitable potential of the four areas of practice derives from the underpinning values that drive the practices. Hence enactments of practice that seek to rework and transform dominant power relations and forms of representation in more substantial ways will support more consequential and longer‐lasting youth outcomes. We also suggest that the equitable potential of the practice of respecting and valuing young people's identities in STEM may be heightened when enacted through loving/caring pedagogy. As discussed next, we suggest that our analyses make contributions in two main areas: (i) advancement of sociological/Bourdieusian theory and (ii) implications for ISL policy and practice.

### 5.1 Contributions to Bourdieusian educational theory

We sought to not just use, but also extend and explore the limits of Bourdieu's conceptual tools for understanding practice and outcomes in relation to young people participating in ISL in the two programs. We found the framework useful and productive in that it helped to identify and explain a key underpinning feature that enabled the equitable potential of the four practices, namely the shifting of dominant power relations and doxa. Understanding youth experiences and outcomes as produced through interactions of habitus, capital, and field also helped explain how and why the same practices were differentially experienced (and produced differential outcomes) between the four focal youth. We suggest that our findings lend further support to existing arguments that the equitable potential of ISL might be fostered by making changes to practice within the field, rather than seeking to change youth. Many mainstream ISL and STEM interventions seek to change the STEM views, attitudes, aspirations, and understanding of young people; however, our research suggests that these might be usefully re‐orientated to focus on changing the field rather than the young person.

However, our analyses also challenged and extended a Bourdieusian interpretation in a number of ways. First, we suggest that some of the pessimism associated with Bourdieusian theory may be overplayed, as our data identified that it is possible for educational fields to support and help challenge—not just to act as an engine of reproduction of—social inequalities. Moreover, the four youth all experienced some form of equitable outcome from their participation—even if these outcomes were modest and transient (given that our data only extend to 4/5 months post‐participation).

Second, our analysis found that these positive outcomes were not purely due to the young people concerned about having dominant forms of capital (as per Bourdieu & Passeron, 1979). Indeed, we identified examples of underserved young people benefitting in meaningful ways from equity‐orientated practices enacted by ISL professionals, like Cole. That is, equitable outcomes were not achieved solely on the basis that the youth in question possessed higher volumes of capital that were recognized by the field. Rather, the programs and ISL practitioners themselves were able to make a difference. The implication of this is that, counter to a Bourdieusian reading, not all institutionalized pedagogic practices constitute pedagogic work in service of the reproduction of elite values and interests. This observation raises the question as to how and why these counter‐hegemonic spaces exist and persist? This is an issue that deserves further consideration and exploration. One possibility is that Bourdieu's theory does not consider the values and practices of educators from minoritised communities—indeed, it has been argued that Bourdieu's theory “cannot account for the nuanced practices of those who do not operate from a dominant position” (Skeggs, 2004, p. 30), such as Cole, whose positionality may support his potential for reimagining normative ISL practice. Existing Bourdieusian work has focused on identifying transformation at the level of the individual habitus and detailing the conditions required for individuals to develop a reflexive habitus. Our analyses identify a potential new area for consideration, namely the capacity for institutions to enact equitable practices and cultures, which might be indicative of the potential for a reflexive institutional habitus that challenges social reproduction and can support the achievement of individual equitable outcomes.

Third, we suggest that the paper posits a broader view of the potential for social justice, as achievable not just through the potential of rational pedagogy (Bourdieu & Passeron, 1979) to support the individual accumulation and social redistribution of capital (although we did identify aspects of this within our data, as young people from underserved communities accrued capital through participation), but through a broader suite of practices (such as the four identified), that seek to challenge power relations within and across fields. Moreover, we note that the critical outcomes achieved were not solely due to pedagogies of critical consciousness‐raising, as espoused by theorists such as Freire (1970)—although again, we noted aspects of this within enactments of the practice of designing for the agency.

Of course, Bourdieu might counterargue that our interpretations are misguided and akin to false consciousness and that the equitable youth outcomes identified are merely transient, partial individual gains that fail to change wider relations of privilege and domination and fail to appreciate how such programs merely reinforce the hegemony of STEM and reflect the paradox of the dominated (Bourdieu 1990c), in which attempts to value identities and experiences that are normally marginalized may not lead to any change in material circumstances whereas social mobility may depend on submission to playing the “game” of the dominant. However, we maintain a belief in the importance and value of practice that challenges and broadens dominant doxa and supports Other ways of thinking and doing, not least within a powerful and elite field such as STEM. Finally, our analyses draw attention to the limitations of Bourdieusian theory for understanding and appreciating the importance of equitable, caring relationships between youth and educators. For this, we argued that it is useful to go beyond Bourdieu's canon, particularly to draw on Black feminist and critical educational work.

### 5.2 Implications for ISL theory and practice

We extrapolate from our analyses that equitable practice may be beneficial for *all* young people—including those, like Star, who may not explicitly enjoy all aspects of the experience and/or whose engagement in more traditional classroom settings tends to be more closely aligned with prototypical STEM education. The equity orientation of an informal science learning program will make an important difference to the potential outcomes and possibilities that it can offer for youth. That is, the equitable potential of an activity or program will be determined by its underpinning norms and values and, crucially, the extent to which it challenges or reproduces the dominant power relations that structure, inform, and mediate social life. It is this which can make a key difference for youth outcomes. This echoes the Florian and Black‐Hawkins' (2011, p. 814) argument that “inclusive pedagogy is defined not in the *choice* of strategy but in its *use*,” emphasis in original). That is, we are not advocating that all ISL settings necessarily adopt, say, creative or outdoors activities as a way to broaden what counts as STEM. Rather, we suggest that practitioners audit their systems and practices to maximize the potential for disrupting and challenging dominant doxa relating to science, youth, and educational relationships through their practice. We further extrapolate, that ISL practices need to be designed with *intentional equity*—that is, foregrounding an explicit recognition and engagement with relations of power and representation to support equitable youth agency and outcomes. This intentional foregrounding is necessary because the default position is not neutral. As Bourdieu reminds us, educational practice as usual entails the reproduction of social privilege. In this respect, we argue that equity issues need to be mainstream, not peripheral, concerns within education programs—with social justice being designed into the aims, goals, delivery, and evaluation of all aspects of all programs.

While our study focuses on ISL, some of the points raised, and indeed the four practices identified may also have resonance for formal STEM education. This is a point that the youth themselves urged consideration of both explicitly and implicitly, as exemplified by an exchange between Lulabelle and Rhubarb observed during one of the sessions, when Lulabelle reflected aloud “why can't science at school be like this?”, to which Rhubarb replied “exactly!”. We recognize of course that this sort of work requires the time, space, and resource to support practitioners' critical reflection and enhancement of practice. To this end, we are aware that more support might usefully be provided to settings and educators (in formal and informal settings) to engage with issues of equity. Currently in England, such opportunities are not an extensive or core feature within the training and/or development of many informal STEM educators, we consider this to be urgently deserving of change.

Traditionally, ISL has tended to cater to those with pre‐existing STEM interests—that is, many ISL participants choose to participate because they are already interested in STEM. However, as exemplified by the four youth, our findings also suggest that valuable outcomes can be achieved through specially designed programs that seek to also engage those who traditionally have not been well‐served by STEM and/or the informal sector. We thus suggest that the sector might usefully focus on further directly supporting and engaging with youth from communities that are traditionally underrepresented in STEM.

The Bourdieusian lens also lends further weight to the importance, as exemplified in the critical ISL literature, of longer‐term interventions for supporting equitable youth outcomes. Bourdieu emphasizes that habitus and capital are developed in and over time, through long‐term processes of socialization and experience. Hence longer term initiatives are more likely to have the capacity to meaningfully support the habitus and capital of youth and to enable the deconstruction of doxa through sustained enactments of the four areas of practice. Loving/caring relationships are also more likely to strengthen and support equitable youth outcomes over longer periods of time. In this respect, the two relatively short‐term programs examined in this paper (lasting just 1 week and a couple of months, respectively) might be judged as falling short in this respect. We thus suggest the ISL sector might give further consideration to supporting more intensive, longer‐term youth programs, rather than the current model of relatively short‐term interventions.

The two programs examined in this paper managed to enact some aspects of the areas of practice outlined by the critical ISL literature, but they also fell short of the ideals in various ways. We suggest that this is perhaps not surprising given that they are programs that are reasonably typical of the wider sector and did not have the same forms of funding or long‐term research—practice partnerships and involvement of ISL equity experts that programs such as GET City have benefitted from. We suggest that these points may also be useful for the sector in continuing to develop its equity potential—and for funders considering how they might best support equitable outcomes from ISL. From a Bourdieusian perspective, such factors are particularly important considerations because the institutions and organization of social life are set up to protect and further the interests of the dominant classes, hence “everyday” practices will be orientated to trying to close down transgressions, rather than support them. In this way, we suggest that it is important to identify the specificity of contexts in which equitable practice is happening to work out the ways in which the field needs to change to enable the enactment of equitable practice. Decontextualized practices cannot simply be picked up and simply applied to different contexts. Hence, we hypothesize that to further enable equitable practices within our two focal settings, the task is not only to further enhance practitioner understanding of the four areas of practice identified by the literature. Fundamental changes to the respective fields will also be needed to enable such practices to be enacted and embedded—and resistant to the ongoing external impetus of social reproduction. From a Bourdieusian perspective, this would require a fundamental reordering of the values, capital, and relations of power that structure each ISL setting. Indeed, the Bourdieusian lens emphasizes how symbolic power depends on having “obtained sufficient recognition to be in a position to impose recognition” (Bourdieu, 1989, p. 23). In other words, the potential for ISL to enact the four areas of equitable practice will depend on those involved having sufficient power and authority to consecrate new legitimate forms of STEM identity, recognition, and practice. We interpret this point as suggesting that for equitable practice to be both effective and mainstreamed within ISL, the sector will need to experience some substantial and fairly radical shifts in power and resource—away from corporate and dominant interests, toward equity practitioners and youth. Otherwise, such efforts will be hindered and the impact will remain constrained. Such changes require “a radical transformation of the social conditions of production” (Bourdieu, 2001, pp. 41–42). Or as Lawler explains, “a radical change to the composition and distribution of symbolic capital would require, as Bourdieu (2001; Bourdieu & Wacquant 2002) suggests, a symbolic revolution, in which ways of knowing, understanding, and perceiving were overturned” (Lawler, 2011).

The Bourdieusian lens thus underlines the scale and political nature of the challenge. The power of the four practices lies in their orientation toward dismantling and interrupting the dominance of ruling classes—reminding us that equity work is not a neutral practice in which “everyone wins.” Supporting equitable youth outcomes will necessarily involve disrupting elitism and privilege.

### 5.3 Limitations

The data discussed are drawn from two specific programs conducted at two ISL settings in England and therefore have very limited scope for offering generalized claims to other settings and international contexts. Both settings were located in London, which necessarily restricts the applicability of findings more widely. For instance, while small city farms and zoos (comparable to the Community Zoo) may be common in many urban areas in the United Kingdom (and some international contexts), they are found less in rural areas. The Girls STEM Club setting offers a more generalizable context, given that it is part of a wider national program of clubs that are held in a range of venues, including schools and community centers across rural and urban locales. Similar clubs can be found in other national contexts, such as the United States. The ethnic diversity among the young people who participated was reflective of the demography of London and hence is not representative of the country as a whole. However, we hope that the interpretations offered can offer useful insights to wider ISL practice in terms of the potential for *framing* practice in more equitable ways.

Our methodology offered limited scope for identifying wider and/or longer‐term potential outcomes. That is, most of the outcomes discussed are necessarily restricted to young people's experiences on the programs. Some examples were noted in relation to longer‐term outcomes that were found to have persisted at the time of the 5‐month follow‐up interviews (notably Star and Lulabelle's eco‐agency, Crystal's confidence, and Innocent's feelings of Black female STEM empowerment). However, we did not expect, nor attempt to collect, evidence of wider outcomes, such as relating to the young people's experiences of school science education or changes in dominant representations of STEM beyond the programs. In this respect, our analyses are necessarily limited, although the endurance of some individual‐level outcomes beyond the programs hints at some useful possibilities for future work to explore and identify the conditions and practices that support the translation and achievement of equitable outcomes across different fields.

## 6 CONCLUSION

This paper has sought to add to understanding about the potential for ISL programs to support equitable youth outcomes. Through the application of a Bourdieusian theoretical lens in dialogue with the critical ISL literature, we identified four enactments of practice that can support equitable outcomes among young people and which were identified to greater or lesser extents in the programs studied. We highlighted how the equity potential of these practices was underpinned by a shifting of the field (rather than focusing on changing the youth), notably by challenging dominant power relations and disrupting STEM doxa. Affordances, limitations, and extensions of the Bourdieusian lens were identified and we argued that while the lens can offer new and useful insights, it needs to be used in conjunction with other critical lenses, not least to capture the importance of equitable and caring relationships between educators and youth. We conclude that equity and social justice need to be explicit, core, intentional goals and values within informal science learning contexts, if they are to support equitable outcomes for youth, but particularly those from historically underrepresented communities.

## APPENDIX 1 ADDITIONAL DETAIL ON SESSION PROGRAMS AND ACTIVITIES

**Zoo program—Example daily schedule**

**Table:**

**Day**	**Session**	**Example activities**
1	Welcome, an overview of programme, introductions, young people and facilitator co‐construct group ground rules	Staff and youth introduce themselves. Practitioners explain the aims and nature of the programme, what will happen and where. Address any questions and concerns
	Morning session: Introduction to evolution	Mixture of practitioner‐led input, class discussion, and portfolio work. Youth are given a selection of photos of birds and are scaffolded to identify and discuss beak and feet adaptions. Youth stick photos in their journals and write notes from their discussions and identifications (e.g., beaks that are good for scavenging vs. eating seeds). Group discussion, led by practitioner, about animal species, kingdoms, and families. Object handling as part of discussions (e.g., youth explore different bird skulls, feathers from the zoo collection). Make notes in journals of learning.
	Lunch	Informal chat
	Afternoon Activity 1: Adaption and enrichment (making feeding tubes for the Ibis)	Discussion about zoos, ethics of zoos, and how we can study habitats in the artificial zoo environment. How to keep animals happy in zoos? (practitioner scaffolds young people to explore the importance of enrichment and ideas for what forms it could take in the zoo)
		Practitioner‐led introduction to the Ibis, one of the birds in the zoo, explaining species, observing key physical features, and which of these features are adaptions to its natural habitat (how/why).
		Young people play a game in which they have to use tweezers to pick up different sorts of bird feed (to work out which sorts of food are the tweezers best suited to picking up?)
		Practitioner‐led recap and discussion about different birds' beaks and the notion of enrichment for zoo animals—how enrichment can involve replicating their habitat and feeding habits in the wild in the zoo enclosure.
		Young people construct feeding tubes for the Ibis in the zoo, constructing them from wood and pipes and filling them with sawdust and live meal worms. They have to discuss and experiment with the implications of filling the tubes to all the same or different heights (What would most closely replicate the natural habitat? Will the birds be able to reach the food? What would provide sufficient challenge/interest?) Completed pipes are put into the ibis enclosure. Recap and summary of what they have covered and learned about adaption and birds beaks. Notes in journals.

	Afternoon Activity 2: Eggs and bird box making	Looking at different eggs (a range of sizes, from different birds). Practitioner facilitates a discussion about how long they think each takes to hatch and which bird it comes from. Facilitator explains the egg cycle. Young people share their own experiences of finding/seeing bird eggs (e.g., in the garden, park). Young people are provided with materials to make/decorate their own bird boxes, which are then put into the zoo enclosures for birds to nest in. Young people consider what decorations would be best (e.g., bright or muted/to fit with the environment) and also personalize the boxes (so they can monitor whose boxes are used by the birds).
	Close/wrap up	Young people are asked to fill in their portfolios and to take them home to add pictures and thoughts on their own interests and lives (i) in general and (ii) anything they particularly like/are interested in relation to nature and science.

**Girls STEM Club—Example half‐term session program**

**Table:**

**Week**	**Session topic**	**Example activities**
1	Maths	Focus on Maryam Mirzakhani (Iranian mathematician and Professor of Mathematics at Stanford University)—watch short YouTube clip about her and group discussion/questions/thoughts about her.
		Members choose either to make an origami paper cube (and upload/share photos with others) or make a poster (that they present to club members at the end) in which they research women mathematicians and make a case for who they think deserves to with a Field medal and why.
		Post any career questions to online STEM role model “Agony aunts”
2	Space travel	Focus on Helen Sharman, the first British female astronaut. Watch short YouTube video about her, followed by discussion and questions.
		Members choose either to recreate space walk challenges experienced by astronauts (wearing multiple pairs of gloves, trying to complete a paper jigsaw puzzle, discussing the properties needed for space gloves, temperature in space, and physical challenges of fine motor movements when in bulky space gear) or conduct online research to create an infographic considering what resources and conditions would be needed for a human return trip to Mars.
		Sharing of activities via social media, questions/discussion.
3	Computer science	Focus on Muffy Calder, computer scientist and engineer. Watch short YouTube video, discussion and Q&A.
		Members choose either to play the 20 questions game with each other (working out the minimum number of yes/no questions it takes to find the answer to a particular question, relating this to computing and what sorts of questions yield the most information; or generate codes in pairs and take turns to decipher coded messages.
		Discussion about importance of universal symbols and what makes codes easier/harder to crack.
4	Aerodynamics	Focus on Nike Folayan, chartered engineer and electronic engineer. Watch a short YouTube clip.
		Members either: work in small groups and make paper aeroplanes which they test to see whose can fly the furthest, modifying wings and body each round. After five rounds, find a winning design.
		Assess what properties of the aeroplane allowed it to out fly the others.
		Or—make an infographic about three engineering companies.
		Include interesting information, for example, their mission and locations.
5	Solar system	Focus on Katherine Johnson, watch YouTube clip about her life and the film Hidden Figures.
		Members choose to either: make a sweet solar system, researching to create a scale model of our solar system, working out the relevant sizes of the planet (and translating into the size of confectionary) and relative distance needed between each planet. Or—work in small groups to create a wall display on the eight major phases of the moon and why they occur (which is presented to the rest of the club).
	Industry visit (school day trip)	STEM club members take part in a school visit (organized by the Girls STEM social enterprise, also involving girls from another school) hosted at a City Tech Consultancy. The day starts with welcome and introductions (staff and students), ice‐breaker activities, an overview of the day. Guided tour of the organization with presentations and short video in the “ideas room.” Introduction to three STEM industry specialists from the host company, who introduce themselves, their interests, backgrounds, and careers (with Q&A).
		The main part of the day is spent coding—practitioners provide an introduction to coding, explanation of the task (to design and build an app to organize a music festival). Girls work in pairs (with a partner from the other school). Staff circulate and support as needed. At the end of the day, the pairs present their festivals and apps to the whole group and apps are judged by the industry experts.
	Hackathon	Optional weekend 2‐day coding event, held at City HQ of multinational investment banking company. Girls can attend either on their own or with their families/carers. Free food and drink is provided for all.
		Arrival, introductions, and ice‐breakers. Short promotional films shown (focusing on the importance of issues of women in STEM, advances in computing), staff from organizing team and the bank are introduced.
		Participants choose whether to go in the Scratch or Python rooms. Support is provided by a range of staff and volunteers. Those using scratch work individually or in small groups to create a digital animation on a topic of their choice. Those in the Python room create a chatbot
		Throughout the day, small groups are taken out to have a go on a Do‐bot, programming a robotic arm and pen to draw a shape—with a prize at the end for the best drawn shape.
